# The correlation of *microRNA-499* rs3746444 T>C locus with the susceptibility of gastric cancer: from a case–control study to a meta-analysis

**DOI:** 10.1042/BSR20203461

**Published:** 2021-01-06

**Authors:** Guoxiang Rong, Yongping Zhu, Weifeng Tang, Hao Qiu, Sheng Zhang

**Affiliations:** 1 Department of Thoracic Surgery, The People’s Hospital of Danyang; Affiliated Danyang Hospital of Nantong University, Danyang, Jiangsu Province, China; 2 Department of Cardiovascular Surgery, Fujian Medical University Union Hospital, Fuzhou, Fujian 350005, China; 3 Department of Cardiothoracic Surgery, Affiliated People's Hospital of Jiangsu University, Zhenjiang, Jiangsu Province, China; 4 Department of Immunology, School of Medicine, Jiangsu University, Zhenjiang, Jiangsu Province, China; 5 Department of General Surgery, Changzhou No. 3 People’s Hospital, Changzhou 213000, Jiangsu Province, China

**Keywords:** gastric cancer, MicroRNA-499, polymorphism

## Abstract

The relationship between rs3746444 T>C single-nucleotide polymorphism (SNP) in *microRNA (mir)-499* and risk of gastric cancer (GC) has been widely investigated. However, the association was still unconfirmed. Here, we first recruited 490 GC patients and 1476 controls, and conducted a case-control study. And we did not find any association between rs3746444 T>C SNP polymorphism and risk of GC. Subsequently, we conducted a meta-analysis to explore the association of *mir-499* rs3746444 polymorphism with GC development. Two authors searched the PubMed and EMBASE databases up to October 15, 2019 independently. Finally, nine literatures involving 12 independent studies were included. In total, 3954 GC cases and 9745 controls were recruited for meta-analysis. The results suggested that allele model, homozygote model and recessive model could increase the risk of overall GC (*P* = 0.002, 0.009 and 0.013, respectively). When we excluded the studies violated HWE, this association was also found in allele model (*P* = 0.020) and dominant model (*P*= 0.044). In subgroup analyses, we identified that rs3746444 SNP in *mir-499* increased the risk of GC in Asians and gastric cardiac adenocarcinoma (GCA) subgroups. No significant bias of selection was found (all *P*>0.1). Test of sensitivity analysis indicated that our findings were stable. Additionally, we found that the power value was 0.891 in the allele model, suggesting the reliability of our findings. In summary, our analysis confirmed the association between rs3746444 and the risk of GC, especially in Asians and in patients with GCA.

## Introduction

Gastric cancer (GC), a commonly malignant disease, which ranks the fifth in terms of cancer diagnose but the third in terms of cancer death (almost one in every twelve mortalities globally) [1]. Compared with other cancers, GC poses both higher incidence and more frequent mortality. Generally, there are two subtypes of GC diagnosed involving gastric cardiac adenocarcinoma (GCA) and non-GCA. The underlying etiology in the development of GC involves the potential interaction between environmental and individual’s genetic factors. A number of efforts have been performed before people make a clear knowing of how the hereditary factors could influence the onset of GC.

In human, *microRNA-* (*mir-*) has about 22 nucleotide molecules. Recently, many types of mir- have been found. It is established that mir- regulates the expression of the target genes. Accumulating evidences have indicated that mir- is very important for adjusting and controlling the various functions in body. Abnormal expression of mir- may lead to a variety of disorders. *Mir-499* is a common *mir-* and extensively studied its potential role in the development of cancer. More and more evidences have indicated that *Mir-499* plays a vital role in growth and migration [2,3], inflammatory response [4], and immune response [5]. A recent study reported that *miR-499-5p* might facilitate the progress of colorectal carcinoma and could be considered as a therapeutic target for the treatment [2]. The *miR-499* signature from the serum was identified to be correlated with prognosis of lung carcinoma [6]. Compared with tubular adenocarcinoma, the *miR-499-3p* expression was increased in signet-ring cell of GC [7]. In view of these, we concluded that *mir-499* could be implicated in carcinoma development.

Single-nucleotide polymorphisms (SNPs) may be the most frequent mutation. *Mir-*SNPs could affect the normal expression of *mir-*. Of late, some studies have sought the relationship of loci in *mir-499* with cancer. Rs3746444 T>C SNP is located in *mir-499* and extensively studied the association of this SNP with cancer risk. Meta-analyses have established the correlation between this SNP and the risk of overall cancer [8–13]. Most of these findings suggested that rs3746444 C-allele carriers appeared to get an increased susceptibility of cancer [8–10,12,13]. The relationship between rs3746444 SNP and GC susceptibility has also been investigated [14–22]. However, the correlation of rs3746444 polymorphism with GC development was still unconfirmed. Additionally, more recent studies with large sample sizes focusing on the relationship between *mir-499* rs3746444 SNP and GC risk have been conducted [15,21]. The potential association of *mir-499* rs3746444 SNP to GC is more conflicting. On this issue, it is necessary to carried out a more precise assessment. Thus, considering the effect of rs3746444 SNP on the risk of GC, we first recruited 1966 subjects (490 GC patients and 1476 controls), and conducted a case–control study. Subsequently, an updated pooled-analysis was carried out to clarify the role of rs3746444 SNP on the development of GC.

## Materials and methods

### Case–control study

In this investigation, 490 histopathologically confirmed GC cases were enrolled from Union Hospital (Fuzhou city, China) and the No.1 People’s Hospital of Zhenjiang City (Zhenjiang City, China), between May 2013 and June 2016, and 1476 hospital-based controls were also recruited as we mentioned in our previous study [23]. All GC patients were diagnosed as non-GCA cases. Age and sex were full-matched in two groups. The information of the included subjects was summarized in Table 1. Each participant provided an informed consent. The institutional review boards of Jiangsu University approved this study protocol (No. 20150083). *Mir-499* rs3746444 SNP was selected to studied. The related information of *mir-499* rs3746444 SNP was summarized in Table 2. By using the Promega DNA Purification Kit (Madison, U.S.A.), genomic DNA was carefully extracted from peripheral blood samples. Genotyping was conducted by SNPscan™ methodology (Genesky Biotechologies Inc., Shanghai, China).

**Table 1:** Distribution of selected demographic variables and risk factors in GC cases and controls

Variable	GC Cases (*n*=490)	Controls (*n*=1476)	*P*^a^
	*n* (%)	*n* (%)	
Age (years)	60.65 ±11.43	61.30 ±9.60	0.220
Age (years)			0.597
< 61	221(45.10)	686(46.48)	
≥61	269(54.90)	790(53.52)	
Sex			0.891
Male	331(67.55)	1,002(67.89)	
Female	159(32.45)	474(32.11)	
Smoking status			**0.001**
Never	309(63.06)	1051(71.21)	
Ever	181(36.94)	425(28.79)	
Alcohol use			**<0.001**
Never	374(76.33)	1319(89.36)	
Ever	116(23.67)	157(10.64)	
BMI (kg/m^2^)			
< 24	356(72.65)	761(51.56)	**<0.001**
≥ 24	134(27.35)	715(48.44)	

Bold values are statistically significant (*P*<0.05).

BMI, body mass index.

* Two-sided *χ*^2^ test and Student’s *t* test.

**Table 2:** Primary information for *mir-499* rs3746444 T>C polymorphism

Genotyped SNPs	*mir-499* rs3746444 T>C
Chromosome	20
Chr Pos (NCBI Build 38)	3499048
MAF* for Chinese in database	0.15
MAF in our controls (*n*=1476)	0.15
*P* value for HWE† test in our controls	0.99
% Genotyping value	99.64%

*MAF, minor allele frequency.

†HWE, Hardy–Weinberg equilibrium.

The χ^2^-test was conducted to compare the difference in the distribution of genotype frequencies between two groups. SAS 9.4 software (Cary, NC, U.S.A.) was harnessed to analyze the data. The *P* value less than 0.05 was considered as statistically significant.

### Meta-analysis

This meta-analysis was reported following the guideline of Preferred Reporting Items for Meta-analyses (PRISMA) (Supplementary Table S1. The checklist of PRISMA) [24].

Two authors (G. Rong and S. Zhang) searched the PubMed and EMBASE electronic databases up to October 15, 2019 independently. The strategy of literature searching was presented as following: (*microRNA-499* OR *mir-499* OR rs3746444) AND (SNP OR mutation OR variant OR polymorphism) AND (cancer OR carcinoma) and (gastric OR stomach OR esophagogastric junction OR gastric cardiac). References in reviews and the included articles were manually searched and checked the potential data. In literature searching process, there was no language limited.

In this meta-analysis, the included investigations should accord with the selecting criteria: (a) done as a retrospective study or a case–control study; (b) evaluated the association of *mir-499* rs3746444 SNP with GC; and (c) we could extract the original data from the eligible study to get the pooled odds ratios (ORs) and 95% confidence intervals (CIs). The corresponding criteria of exclusion were: (a) repeated data; (b) genotype data were not presented in publication; (c) only focusing on the prognosis of GC; and (d) comments, review and meta-analysis. Two authors (G. Rong and S. Zhang) performed the procedure of data extraction independently. The following original information was selected and extracted: publication year, first author, race, country, number of subjects, method of polymerase chain reaction, *mir-499* rs3746444 genotype data. If any disagreement emerged, the third author (W. Tang) was invited. The final decision was made by a vote during this process.

For *mir-499* rs3746444 SNP, rs3746444 C allele was used as the reference. The relationship was evaluated by using STATA software (version 12.0). We used an online calculator (http://ihg.gsf.de/cgi-bin/hw/hwa1.pl) to determine whether the genotype distribution of *mir-499* rs3746444 agreed with Hardy–Weinberg equilibrium (HWE) [25]. Since the heterogeneity could influence the assessment of association, we used *Q*-statistical test and *I^2^* test to study the heterogeneity. When significant heterogeneity (*I^2^*>50% or *P*<0.10) was found, the random-effects model (the DerSimonian–Laird method) was used [26,27]. And when it is out of heterogeneity, fixed-effects model was implemented (the Mantel–Haenszel method) [28,29]. Four major genetic models were harnessed in the present study. The allele model (C vs*.* T), homozygote model (CC vs*.* TT), recessive model (CC vs*.* TT/TC), and dominant model (CC/TC vs*.* TT) were calculated. Region of GC was defined as gastric cardiac adenocarcinoma (GCA), non-GCA and mixed. Bgger’s Funnel plots and Egger’s test were used to check whether there was an evidence of publication bias. Quality evaluation was carried out with a Newcastle–Ottawa Quality Assessment Scale. The quality of the include study was defined as high quality (scores ≥ 7 stars) and low quality (scores < 7 stars) [30].

## Results

### Case–control study

In total, 1966 subjects (490 non-GCA patients and 1476 controls) were recruited in the present study. The TT, TC and CC genotype frequencies of *mir-499* rs3746444 SNP were 69.82%, 26.69% and 3.49% in 490 non-GCA patients and 71.88%, 25.82% and 2.31% in 1476 hospital-based controls, respectively. As summarized in Table 3, we did not find any association between rs3746444 T>C SNP polymorphism and risk of non-GCA. After an adjustment for the included risk factors, there was no correlation of rs3746444 SNP with the occurrence of non-GCA. The detailed data were summarized in Supplementary Table S2.

**Table 3:** Logistic regression analyses of associations between *mir-499* rs3746444 T>C polymorphism and GC

Genotype	CRC Cases (*n*=490)		Controls (*n*=1476)		Crude OR (95%CI)	*P*	Adjusted OR^*^ (95%CI)	*P*
	*n*	%	*n*	%				
*mir-499* rs3746444 T>C								
TT	340	69.82	1058	71.88	1.00		1.00	
TC	130	26.69	380	25.82	1.07(0.84–1.35)	0.600	1.04(0.82–1.33)	0.730
CC	17	3.49	34	2.31	1.56(0.86–2.82)	0.145	1.59(0.86–2.95)	0.141
CT+TT	147	30.18	414	28.13	1.11(0.88–1.38)	0.384	1.09(0.86–1.37)	0.479
TT+TC	470	96.51	1,438	97.69	1.00		1.00	
CC	17	3.49	34	2.31	1.53(0.85–2.76)	0.159	1.57(0.85–2.90)	0.149
C allele	164	16.84	448	15.22				

* Adjusted for age, sex, smoking status, alcohol use and BMI status.

### Meta-analysis results

**Figure 1:**
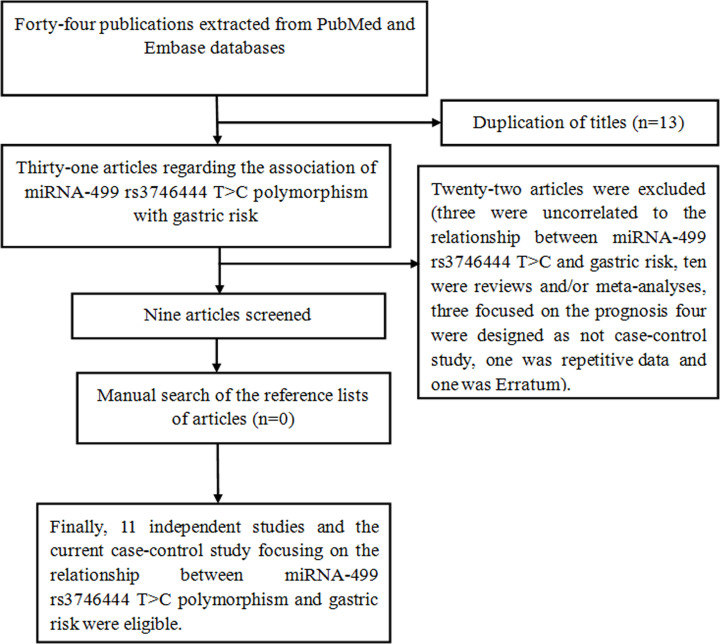
Flow diagram of the meta-analysis

First, 44 literatures were searched from EMBASE and PubMed databases. As shown in Figure 1, when we reviewed the titles and abstracts, 13 duplicated publications were excluded. With an additional filter, twenty-two articles were excluded (ten were reviews and meta-analyses, four were designed as not case–control study, three were uncorrelated to the relationship of rs3746444 with GC risk, three focused on the prognosis, one was repetitive data and one was Erratum). Finally, after a detailed filtrate, nine literatures and the current case–control study involving twelve independent studies were included [14–22]. In total, we recruited 3954 GC cases and 9745 controls. These contained publications were performed in Asians [14,15,17–20] and Caucasians [16,21,22]. Three independent studies were conducted in GCA [15,16], seven were in non-GCA [14,16,18–20,22], and two were in mixed [17,21]. Other detailed information was presented in Tables 4 and 5. According to Newcastle–Ottawa scale, quality assessment of meta-analysis was performed. The process and results of quality assessment were summarized in Table 6.

**Table 4:** Characteristics of the studies in meta-analysis

Authors	Year	Country	Ethnicity	Study design	Number cases/controls	Region of GC	Genotyping method
Rogoveanu	2017	Romania	Caucasians	HB	111/288	Non-GCA	Taqman
Rogoveanu	2017	Romania	Caucasians	HB	31/288	GCA	Taqman
Poltronieri-Oliveira	2017	Brazil	Caucasians	HB	150/239	Non-GCA	PCR-RFLP
Cai	2015	China	Asians	PB	363/969	Non-GCA	MassARRAY
Wu	2013	China	Asians	HB	200/211	Mixed	PCR-RFLP
Pu	2013	China	Asians	HB	196/504	Non-GCA	PCR-RFLP
Ahn	2013	Korea	Asians	HB	461/447	Non-GCA	PCR-RFLP
Okubo	2010	Japan	Asians	HB	552/697	Non-GCA	PCR-RFLP
Torruella-Loran	2019	Ten European countries	Caucasians	PB	365/1284	Mixed	PCR-RFLP
Tang	2019	China	Asians	HB	305/1677	GCA	SNPscan
Tang	2019	China	Asians	HB	758/1677	GCA	SNPscan
Our study	2020	China	Asians	HB	490/1476	Non-GCA	SNPscan

GC: gastric cancer;

GCA: gastric cardiac adenocarcinoma;

H-B: hospital-based;

P-B: population-based;

PCR-RFLP: polymerase chain reaction-restriction fragment length polymorphism.

**Table 5:** Distribution of miR-499 rs3746444 T>C genotypes and alleles

First author	Year	Case TT	Case TC	Case CC	Control TT	Control TC	Control CC	Case C	Case T	Control C	Control T	HWE
Torruella-Loran	2019	244	99	17	834	396	48	133	587	492	2,064	Yes
Tang	2019	199	90	9	1,214	419	41	108	488	501	2,847	Yes
Tang	2019	496	221	25	1,214	419	41	271	1,213	501	2,847	Yes
Rogoveanu	2017	65	44	2	173	107	8	48	174	123	453	Yes
Rogoveanu	2017	15	14	2	173	107	8	18	44	123	453	Yes
Poltronieri-Oliveira	2017	97	48	5	143	90	6	58	242	102	376	Yes
Cai	2015	261	89	13	765	179	25	115	611	229	1,709	No
Wu	2013	149	47	4	166	42	3	55	345	48	374	Yes
Pu	2013	141	50	5	366	121	17	60	332	155	853	Yes
Ahn	2013	323	123	15	299	134	14	153	769	162	732	Yes
Okubo	2010	364	151	37	466	198	33	225	879	264	1,130	No
Our study	2020	340	130	17	1,058	380	34	164	810	448	2,496	Yes

HWE, Hardy–Weinberg equilibrium

**Table 6:** Quality assessment of the meta-analysis

Study	Year	Selection				Comparability of the cases and controls	Exposure			Total Stars
		Adequate case definition	Representat- iveness of the cases	Selection of the controls	Definition of Controls		Ascertainment of exposure	Same ascertainment method for cases and controls	Non- response rate	
Rogoveanu	2017	★	★	-	★	★★	★	★	-	7
Rogoveanu	2017	★	★	-	★	★★	★	★	-	7
Poltronieri-Oliveira	2017	★	★	-	★		★	★	-	5
Cai	2015	★	★		★		★	★	-	5
Wu	2013	★	★	-	★	★★	★	★	-	7
Pu	2013	★	★	-	★	★★	★	★	-	7
Ahn	2013	★	★	-	★	★★	★	★	-	7
Okubo	2010	★	★	-	★	★★	★	★	-	7
Torruella-Loran	2019	★	★	★	★	★★	★	★	-	8
Tang	2019	★	★	-	★	★★	★	★	-	7
Tang	2019	★	★	-	★	★★	★	★	-	7
Our study	2020	★	★	-	★	★★	★	★	-	7

**Figure 2:**
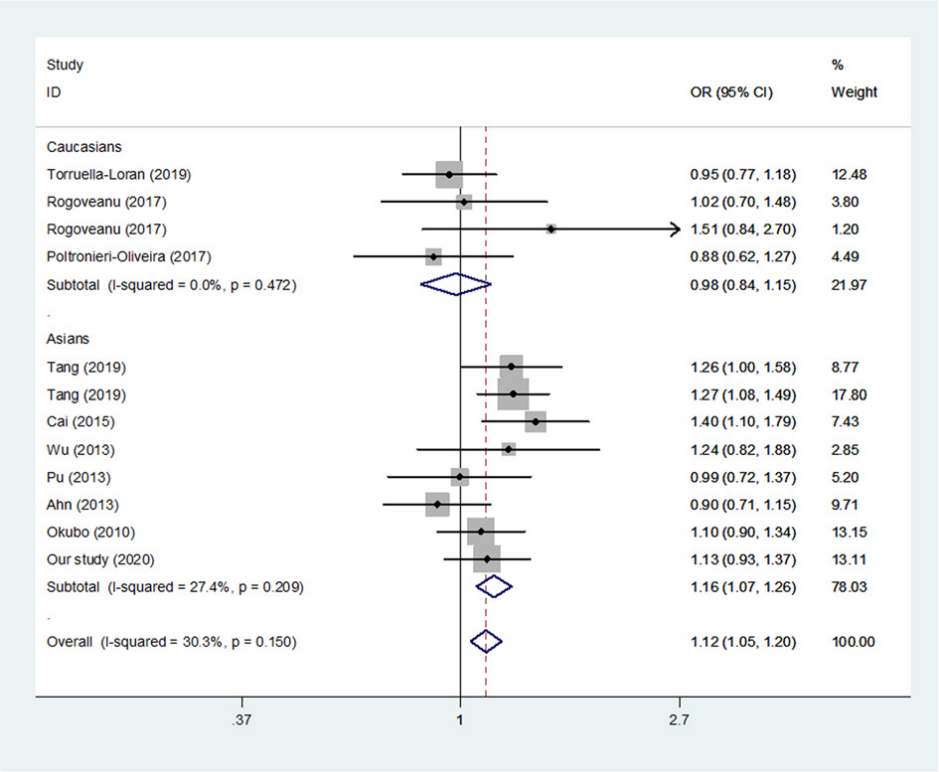
Meta-analysis of the relationship between miR-499 rs3746444 T>C polymorphism and gastric risk for different ethnicity (C vs. T, fixed-effects model)

**Figure 3:**
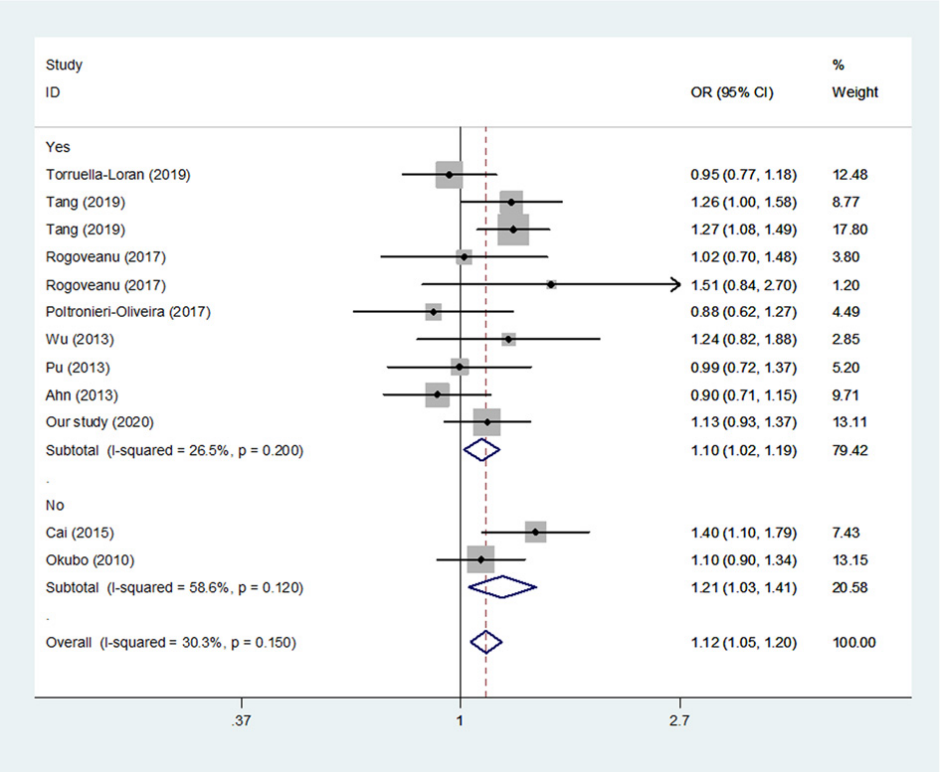
Meta-analysis of the relationship between miR-499 rs3746444 T>C polymorphism and gastric risk for different HWE (C vs. T, fixed-effects model)

The estimated allele model (*P* = 0.002, Table 7 and Figure 2), homozygote model (*P* =0.009), recessive model (*P* =0.013) and dominant model (*P* =0.061) suggested that allele, homozygote and recessive genetic models could increase the risk of overall GC, while the dominant model might not confirm the risk to overall GC. If we excluded the studies violated HWE, this SNP was also found to be correlated with GC susceptibility (C vs. T, *P* = 0.020, CC/TC vs. TT, *P* = 0.044, Figure 3).

**Table 7:** Results of the meta-analysis

	No. of studies	C vs. T				CC vs. TT				CC/TC vs. TT				CC vs. TT/TC			
		OR (95% CI)	*P*	*I*^2^	*P* (Q-test)	OR (95% CI)	*P*	*I^2^*	*P* (Q-test)	OR (95% CI)	*P*	*I^2^*	*P* (Q-test)	OR (95% CI)	*P*	*I^2^*	*P* (Q-test)
Total	12	**1.12(1.05–1.20)**	**0.002**	30.3%	0.150	**1.33(1.07–1.64)**	**0.009**	0.0%	0.964	1.11(1.00–1.24)	0.061	39.4%	0.078	**1.30(1.06–1.60)**	**0.013**	0.0%	0.983
HWE																	
Yes	10	**1.10(1.02–1.19)**	**0.020**	26.5%	0.200	1.28(1.00**–**1.63)	0.054	0.0%	0.917	**1.10(1.00–1.21)**	**0.044**	35.9%	0.121	1.26(0.98–1.61)	0.070	0.0%	0.955
No	2	1.23(0.96**–**1.57)	0.097	58.6%	0.120	1.46(0.98**–**2.18)	0.061	0.0%	0.889	1.23(0.88**–**1.71)	0.232	70.5%	0.066	1.43(0.97–2.12)	0.074	0.0%	0.943
Ethnicity																	
Caucasians	4	0.98(0.84**–**1.15)	0.788	0.0%	0.472	1.21(0.75**–**1.93)	0.437	0.0%	0.651	0.94(0.78**–**1.13)	0.504	0.0%	0.407	1.25(0.78–1.98)	0.356	0.0%	0.713
Asians	8	**1.16(1.07–1.26)**	**<0.001**	27,4%	0.209	**1.36(1.07–1.72)**	**0.011**	0.0%	0.933	**1.17(1.07–1.28)**	**0.001**	35.9%	0.143	**1.32(1.04–1.66)**	**0.020**	0.0%	0.955
Cancer type																	
GAC	3	**1.28(1.12–1.45)**	**<0.001**	0.0%	0.848	1.49(1.00**–**2.24)	0.053	0.0%	0.703	1.32(1.14**–**1.53)	**<0.001**	0.0%	0.872	1.38(0.92–2.07)	0.116	0.0%	0.758
Non-GCA	7	1.08(0.98**–**1.19)	0.121	29.1%	0.206	1.28(0.97**–**1.69)	0.079	0.0%	0.811	1.06(0.95**–**1.19)	0.269	33.4%	0.173	1.27(0.97–1.68)	0.083	0.0%	0.834
Mixed	2	1.01(0.83**–**1.21)	0.961	21.3%	0.260	1.24(0.73**–**2.12)	0.423	0.0%	0.804	0.97(0.78**–**1.20)	0.757	41.0%	0.193	1.29(0.76–2.19)	0.348	0.0%	0.895
Sample sizes																	
<1000	6	0.99(0.86-1.15)	0.930	0.0%	0.529	1.03(0.65**–**1.63)	0.904	0.0%	0.782	0.98(0.84**–**1.16)	0.872	0.0%	0.466	1.04(0.66–1.64)	0.868	0.0%	0.826
≥1000	6	**1.17(1.08–1.27)**	**<0.001**	35.7%	0.169	**1.42(1.12–1.80)**	**0.003**	0.0%	0.992	**1.17(1.02–1.34)**	**0.027**	51.8%	0.066	**1.39(1.10–1.75)**	**0.006**	0.0%	0.997
Source of control																	
H-B	10	**1.12(1.05–1.20)**	**0.004**	11.1%	0.340	**1.33(1.04–1.68)**	**0.021**	0.0%	0.915	**1.12(1.02–1.23)**	**0.014**	20.6%	0.253	**1.30(1.02–1.64)**	**0.031**	0.0%	0.945
P-B	2	1.15(0.78**–**1.69)	0.474	82.3%	0.017	1.33(0.86**–**2.06)	0.204	0.0%	0.613	1.14(0.70**–**1.85)	0.597	85.3%	0.009	1.32(0.86–2.04)	0.208	0.0%	0.828
Quality scores																	
≥7.0	10	**1.11(1.03–1.20)**	**0.007**	16.2%	0.294	**1.31(1.05–1.64)**	**0.018**	0.0%	0.909	**1.11(1.01–1.21)**	**0.025**	26.7%	0.198	**1.29(1.03–1.61)**	**0.024**	0.0%	0.945
<7.0	2	1.14(0.72**–**1.79)	0.580	77.2%	0.036	1.44(0.79**–**2.62)	0.229	0.0%	0.762	1.12(0.63–1.99)	0.705	80.8%	0.022	1.39(0.77–2.51)	0.281	0.0%	0.948

GCA, gastric cardiac adenocarcinoma;

H-B, hospital-based;

HWE, Hardy–Weinberg equilibrium;

P-B, population-based.

In a subgroup analysis for ethnicity, we identified that rs3746444 was associated with the risk of GC in Asians (*P* < 0.05 in all gentic models).

**Figure 4:**
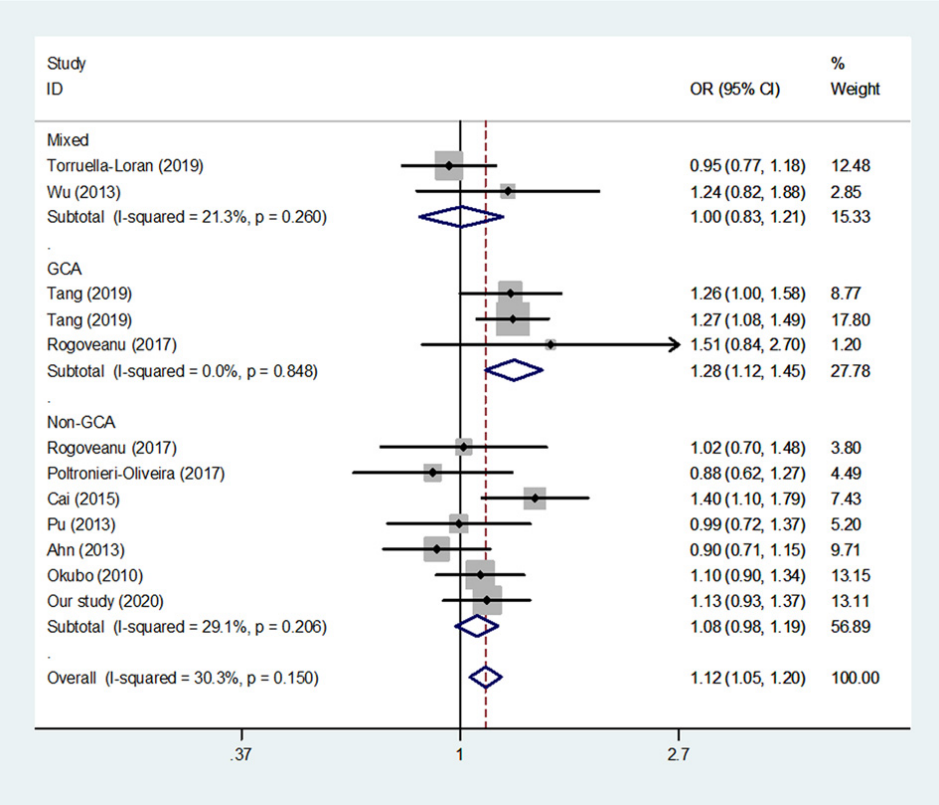
Meta-analysis of the relationship between miR-499 rs3746444 T>C polymorphism and gastric risk for different region (C vs. T, fixed-effects model)

When we conducted a subgroup analysis for region of GC, we found that rs3746444 increased the susceptibility of GCA (C vs. T, *P* < 0.001 and CC/TC vs*.* TT, *P*  < 0.001, Figure 4).

**Figure 5:**
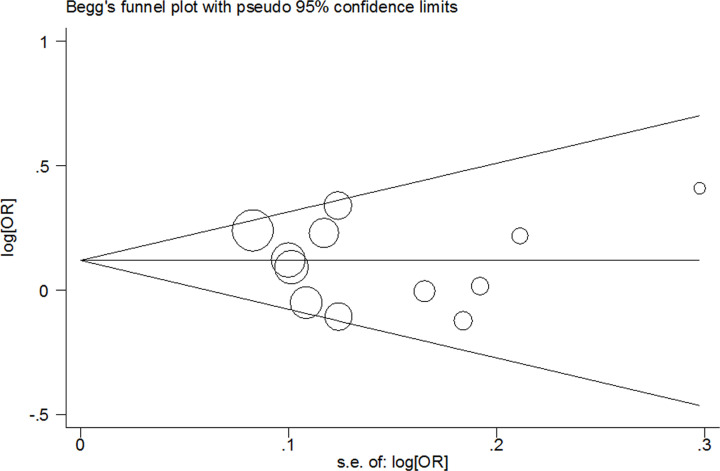
Begg’s funnel plot of meta-analysis (C vs. T, fixed-effects model)

In this meta-analysis, we used Bgger’s funnel plots and Egger’s test to evaluate the potential bias among the included literatures. After viewing Bgger’s funnel plots, symmetrical figure was observed, suggesting no significant bias existing (Figure 5). Egger’s test also indicated that there are no significant bias of selection (all *P*>0.1, data not shown).

In our study, heterogeneity was also evaluated. In dominant model, we identified significant heterogeneity. In order to explore the source of heterogeneity, subgroup analysis were harnessed. In subgroup analysis, we identified an association of the studies violated HWE, Asians, large sample size designed (≥1000 subjects), mixed GC, low quality studies and population-based study subgroups with major heterogeneity.

**Figure 6:**
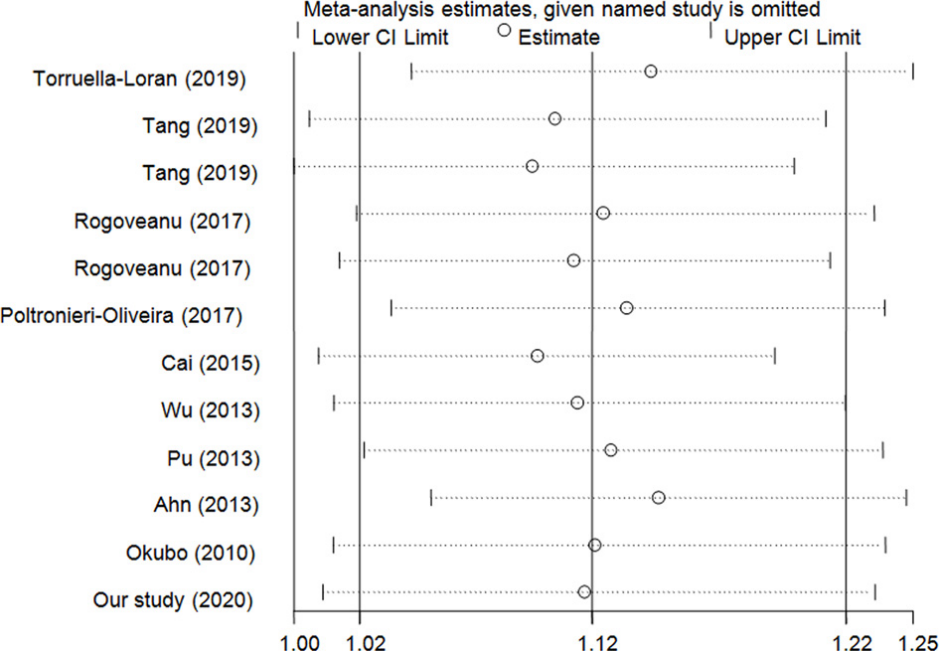
Sensitivity analysis of the influence of C vs. T genetic model (fixed-effects model)

We conducted a sensitivity analysis to assess the stability of the present findings by omitting each study in turn. We calculated ORs and CIs of remainers to evaluate the influence of each study on the overall results. The findings indicated that no individual study could alter the overall assessment significantly (Figure 6), which validated the credibility of these observations.

### Power of meta-analysis (*α* = 0.05)

In the present study, we calculated the power value in the overall comparison. The value was 0.891 in the allele model, 0.766 in the homozygote model and 0.704 in the recessive model.

## Discussion

*Mir-499* may play an important role in the initial and progress of cancer. It was reported that *mir-499-5p* could promote progression of colorectal cancer and might be considered as a therapeutic target [2]. In addition, Qiu et al. identified that the rs3746444 T→C variant might lead to worse survival of lung cancer [31]. And this SNP was a useful cancer biomarker, which was implicated in the development and treatment of lung cancer. The relationship of *mir-499* rs3746444 C-allele carriers with an increased susceptibility of cancer have been identified in some meta-analysis [8–10,12,13]. Of late, meta-analyses have tried to determine the correlation between this SNP and the risk of GC [8–13,32,33]. And all of them have obtained the null association, which maybe due to the limited literatures. A more recent study has focused on the correlation of rs3746444 and GC susceptibility with 2740 subjects [15]. And it reported that rs3746444 significantly increased the susceptibility of GCA. In this case–control study, we first recruited 1966 subjects (490 non-GCA patients and 1476 controls), and did not find any association between rs3746444 T>C SNP polymorphism and risk of non-GCA. Thus, the relationship of rs3746444 SNP in *mir-499* with GC risk was more conflicting. In this meta-analysis, we included 12 case–control studies with 3954 GC cases and 9745 controls to explore the relationship of rs3746444 with GC risk. We first confirmed that rs3746444 SNP in *mir-499* was associate with the susceptibility of overall GC, especially in GCA and Asians subgroups.

In this pooled-analysis, we included 12 independent studies. These literatures were conducted in different races. In view of these studies, the conflicting findings were observed, which made this pooled-analysis interesting and imperative. Cai et al.’s investigation, in eastern China, suggested that the *mir-499* rs3746444 C-allele could promote a susceptibility of GC compared with *mir-499* rs3746444 T allele [14]. Additionally, an another publication reported that *mir-499* rs3746444 C-allele was also associated with the development of GC [15]. However, others could not find any relationship of rs3746444 SNP with GC risk [16–22]. The most vital characteristic of this meta-analysis was that the present study first confirmed the correlation between rs3746444 SNP in *miR-499* and GC development. We also found that this potential association was significant, especially in Asians and GCA subgroups. Wang et al*.* have identified that *mir-499*, by reducing astrocyte elevated gene-1, plays a tumor-suppressive role in the development of hepatocellular carcinoma [34]. A previous study reported that, compared with the subjects with rs3746444 TT genotypes, individuals with *mir-499* rs3746444 TC and CC genotypes have lower expression of miR-499a [35]. It is found that the rs3746444 in mir-499a gene is located on the seed region and affects the arm selection [36]. And as a result, a functional investigation indicated that *mir-499* rs3746444 C-allele decrease the expression of the *mir* [36]. Thus, it is suggested that *mir-499* rs3746444 C-allele promotes a susceptibility of GC by inhibiting the expression of the *mir-499.* It is reported that inclusion of articles violated the HWE may lead to bias. Here, if we excluded these studies, this SNP also conferred a risk to overall GC. It is worth noting that we did not find an association between rs3746444 SNP and non-GCA risk in the current case–control study, which was consistent with the results of meta-analysis in subgroup analyses. The mechanism of GC in different region may be diverse [37–41]. *Mir-499* rs3746444 may play a different role in different type of GC. However, for lack of experimental data, we did not take them into account in the present study. In the future, more functional studies are needed to focus on the potential mechanism.

Two significant problems, publication bias and heterogeneity, should be discussed. No significant publication bias was detected in our study, indicating the dependability of these findings. Between publications for *mir-499* rs3746444 SNP, we only detected a moderate heterogeneity in dominant genetic model. Subgroup analyses suggested an association of the studies violated HWE, Asians, large sample size designed (≥1000 subjects), mixed GC, low quality studies and population-based study subgroups with major heterogeneity. In addition, the power of the present study (*α*=0.05) was also evaluated. We found that the power value was 0.891 in the allele model, suggesting the reliability of our findings.

Despite the present study has pooled all publications and explored the association of rs3746444 with GC development, some limitations also should be addressed. First, only 12 independent case–control studies with 3954 GC cases and 9745 controls were eligible. Second, for lack of some important data (e.g. gender, age, the infection of *helicobacter pylori*, family history of cancer, tobacco using, alcohol consumption and other lifestyles), we only calculated the crude ORs with 95% CIs to determine the relationship of rs3746444 with GC susceptibility. The effect of those factors mentioned above was not taken into account. Thirdly, only the literatures published in English were eligible, which could lead to the bias of selection. Fourth, maybe it would be helpful to validate our findings with an independent cohort. However, due to lack of sufficient data, a cohort study was not performed. Finally, despite we first identified the relationship between rs3746444 and GC development, the function and mechanism of this polymorphism remained unknown.

In summary, our analysis confirmed the association between rs3746444 and the risk of GC, especially in Asians and in patients with GCA. Therefore, more studies are required to explore the potential mechanisms.

## Supplementary Material

Supplementary Tables S1-S2

## Data Availability

Full data are available via an online supplementary material. Supplementary Table S1 summarizes the guideline of Preferred Reporting Items for Meta-analyses. Supplementary Table S2 summarizes the detailed data of genotypes.

## Abbreviations

GC: gastric cancer
GCA: gastric cardiac adenocarcinoma
HWE: Hardy–Weinberg equilibrium
SNP: single-nucleotide polymorphism
