# Supplementary material for: The correlation of microRNA-499 rs3746444 T>C locus with the susceptibility of gastric cancer: from a case–control study to a meta-analysis
Source: Biosci Rep. 2021 Jan 6;41(1):BSR20203461. doi: 10.1042/BSR20203461 (PMC7789807; doi:10.1042/BSR20203461)
Supplement: Supplementary Tables S1-S2 [file BSR-2020-3461_supp.pdf]

PRISMA 2009 Checklist

Section/topic	#	Checklist item	Reported on section #
**TITLE**			
Title	1	Identify the report as a systematic review, meta-analysis, or both.	Title page
**ABSTRACT**			
Structured summary	2	Provide a structured summary including, as applicable: background; objectives; data sources; study eligibility criteria, participants, and interventions; study appraisal and synthesis methods; results; limitations; conclusions and implications of key findings; systematic review registration number.	Abstract page
**INTRODUCTION**			
Rationale	3	Describe the rationale for the review in the context of what is already known.	Introduction, 3rd paragraph
Objectives	4	Provide an explicit statement of questions being addressed with reference to participants, interventions, comparisons, outcomes, and study design (PICOS).	Introduction, 3rd paragraph
**METHODS**			
Protocol and registration	5	Indicate if a review protocol exists, if and where it can be accessed (e.g., Web address), and, if available, provide registration information including registration number.	N/A
Eligibility criteria	6	Specify study characteristics (e.g., PICOS, length of follow-up) and report characteristics (e.g., years considered, language, publication status) used as criteria for eligibility, giving rationale.	Materials and Methods, meta-analysis, 3rd paragraph
Information sources	7	Describe all information sources (e.g., databases with dates of coverage, contact with study authors to identify additional studies) in the search and date last searched.	Materials and Methods, meta-analysis, 2nd paragraph
Search	8	Present full electronic search strategy for at least one database, including any limits used, such that it could be repeated.	Materials and Methods, meta-analysis, 2nd paragraph
Study selection	9	State the process for selecting studies (i.e., screening, eligibility, included in systematic review, and, if applicable, included in the meta-analysis).	Materials and Methods, meta-analysis, 3rd paragraph
Data collection process	10	Describe method of data extraction from reports (e.g., piloted forms, independently, in duplicate) and any processes for obtaining and confirming data from investigators.	Materials and Methods, meta-analysis, 3rd paragraph

Data items	11	List and define all variables for which data were sought (e.g., PICOS, funding sources) and any assumptions and simplifications made.	Materials and Methods, meta-analysis, 3rd paragraph
Risk of bias in individual studies	12	Describe methods used for assessing risk of bias of individual studies (including specification of whether this was done at the study or outcome level), and how this information is to be used in any data synthesis.	Materials and Methods, meta-analysis, 4th paragraph
Summary measures	13	State the principal summary measures (e.g., risk ratio, difference in means).	Materials and Methods, meta-analysis, 4th paragraph
Synthesis of results	14	Describe the methods of handling data and combining results of studies, if done, including measures of consistency (e.g., I^2) for each meta-analysis.	Materials and Methods, meta-analysis, 4th paragraph

Section/topic	#	Checklist item	Reported on page #
Risk of bias across studies	15	Specify any assessment of risk of bias that may affect the cumulative evidence (e.g., publication bias, selective reporting within studies).	Materials and Methods, Statistical methods, 4th paragraph
Additional analyses	16	Describe methods of additional analyses (e.g., sensitivity or subgroup analyses, meta-regression), if done, indicating which were pre-specified.	Materials and Methods, Statistical methods, 4th paragraph
**RESULTS**			
Study selection	17	Give numbers of studies screened, assessed for eligibility, and included in the review, with reasons for exclusions at each stage, ideally with a flow diagram.	Results, Meta-analysis results, 1st paragraph and Figure 1
Study characteristics	18	For each study, present characteristics for which data were extracted (e.g., study size, PICOS, follow-up period) and provide the citations.	Results, Meta-analysis results, 1st paragraph, Table 4 and Table 5
Risk of bias within studies	19	Present data on risk of bias of each study and, if available, any outcome level assessment (see item 12).	Results, Meta-analysis results, 5-7th paragraph, and Figure 5-6 and Table 6.
Results of individual studies	20	For all outcomes considered (benefits or harms), present, for each study: (a) simple summary data for each intervention group (b) effect estimates and confidence intervals, ideally with a forest plot.	Results, Meta-analysis results, 2nd-4th paragraph Figure 2-4 and Table 7
Synthesis of results	21	Present results of each meta-analysis done, including confidence intervals and measures of consistency.	Results, Meta-analysis results, 2nd-4th paragraph

			Figure 2-4 and Table 7
Risk of bias across studies	22	Present results of any assessment of risk of bias across studies (see Item 15).	Results, Meta-analysis results, 5-7th paragraph, and Figure 5-6 and Table 6.
Additional analysis	23	Give results of additional analyses, if done (e.g., sensitivity or subgroup analyses, meta-regression [see Item 16]).	Results, Meta-analysis results, 5-7th paragraph, and Figure 5-6 and Table 6.
**DISCUSSION**			
Summary of evidence	24	Summarize the main findings including the strength of evidence for each main outcome; consider their relevance to key groups (e.g., healthcare providers, users, and policy makers).	Discussion, 1st paragraph
Limitations	25	Discuss limitations at study and outcome level (e.g., risk of bias), and at review-level (e.g., incomplete retrieval of identified research, reporting bias).	Discussion, 4th paragraph
Conclusions	26	Provide a general interpretation of the results in the context of other evidence, and implications for future research.	Discussion, 5th paragraph
**FUNDING**			
Funding	27	Describe sources of funding for the systematic review and other support (e.g., supply of data); role of funders for the systematic review.	Grant support

Subjects	Sex (male: 1; female: 2)	Age (year)	Smoking(Yes : 1, No: 0)	Drinking (Yes: 1, No: 0)	BMI (⩾24: 1, <24: 0)	Rs3746444 genotypes
case-001	2	72	0	0	0	G/A
case-002	1	63	0	0	0	G/A
case-003	1	63	0	0	1	A/A
case-004	1	52	1	0	0	A/A
case-005	1	66	1	1	0	A/A
case-006	1	49	0	0	0	G/A
case-007	2	54	0	0	1	A/A
case-008	2	44	0	0	0	A/A
case-009	1	71	1	0	0	A/A
case-010	1	50	1	0	0	A/A
case-011	1	69	0	0	1	A/A
case-012	1	53	0	0	0	A/A
case-013	1	83	0	0	0	G/A
case-014	1	83	0	0	1	G/A
case-015	1	48	0	1	0	A/A
case-016	1	65	1	0	0	A/A
case-017	1	69	0	0	1	G/A
case-018	1	77	0	0	0	A/A
case-019	2	53	0	0	0	G/A
case-020	1	48	1	0	0	G/A
case-021	2	52	0	0	1	A/A
case-022	1	46	0	0	0	A/A
case-023	2	69	0	0	0	A/A
case-024	1	67	1	0	0	G/A
case-025	1	68	1	0	1	?
case-026	1	61	1	0	0	G/A
case-027	1	67	1	1	0	A/A
case-028	2	44	0	0	0	A/A
case-029	1	78	1	0	0	G/A
case-030	2	29	0	0	0	G/A
case-031	1	70	1	1	0	A/A
case-032	1	84	0	0	0	A/A
case-033	2	61	0	0	0	A/A
case-034	1	65	0	0	1	G/A
case-035	2	66	0	0	0	A/A
case-036	1	75	1	1	0	A/A
case-037	1	64	0	0	0	G/A
case-038	2	64	0	0	0	G/A
case-039	1	76	0	0	0	G/A
case-040	1	55	1	1	1	A/A
case-041	1	57	1	1	1	A/A
case-042	1	50	0	0	1	A/A
case-043	1	70	0	0	0	A/A
case-044	2	54	0	0	0	A/A
case-045	1	49	0	0	1	A/A
case-046	2	53	0	0	0	A/A
case-047	1	72	0	0	1	A/A
case-048	2	64	0	0	0	A/A
case-049	1	57	1	1	0	G/A
case-050	2	52	0	0	1	A/A
case-051	2	69	0	0	1	A/A
case-052	2	71	0	0	0	A/A
case-053	1	78	0	0	1	A/A
case-054	2	51	0	0	0	A/A
case-055	2	33	0	0	0	A/A

case-056	1	64	1	0	0	A/A
case-057	1	51	0	0	0	A/A
case-058	1	78	1	0	1	A/A
case-059	1	79	1	0	0	G/A
case-060	2	77	0	0	1	A/A
case-061	1	38	1	0	1	A/A
case-062	2	54	0	0	0	A/A
case-063	2	44	0	0	0	A/A
case-064	1	72	0	0	1	A/A
case-065	1	82	0	1	0	A/A
case-066	2	69	0	0	0	G/A
case-067	2	44	0	0	0	G/A
case-068	1	59	1	1	0	G/A
case-069	2	78	0	0	0	A/A
case-070	1	57	1	1	1	A/A
case-071	2	45	0	0	0	A/A
case-072	1	50	1	1	0	A/A
case-073	1	57	0	0	0	A/A
case-074	1	65	1	1	0	A/A
case-075	2	51	0	0	1	G/G
case-076	1	67	1	1	1	A/A
case-077	2	67	0	0	0	A/A
case-078	1	50	1	1	0	A/A
case-079	1	64	1	0	0	A/A
case-080	1	54	1	0	0	A/A
case-081	1	42	0	1	0	A/A
case-082	1	75	1	1	0	G/A
case-083	1	72	1	1	0	A/A
case-084	2	56	0	0	0	G/A
case-085	1	50	1	1	0	G/A
case-086	1	66	1	1	0	G/A
case-087	1	42	0	1	0	A/A
case-088	1	41	0	1	0	A/A
case-089	1	50	1	1	0	A/A
case-090	1	60	0	0	0	A/A
case-091	1	66	1	1	0	A/A
case-092	2	31	0	0	1	A/A
case-093	1	73	0	0	0	A/A
case-094	1	42	1	0	0	A/A
case-095	1	50	1	0	0	A/A
case-096	2	29	0	0	0	A/A
case-097	2	41	0	0	0	G/A
case-098	2	49	0	0	1	G/A
case-099	2	42	0	0	0	G/A
case-100	1	54	1	1	0	G/A
case-101	2	53	0	0	1	G/A
case-102	1	62	1	1	0	G/A
case-103	2	44	0	0	0	A/A
case-104	2	61	1	0	1	A/A
case-105	1	74	1	0	0	A/A
case-106	1	59	0	0	0	G/A
case-107	2	34	0	0	0	A/A
case-108	1	62	0	0	0	A/A
case-109	1	60	1	0	1	A/A
case-110	1	80	1	1	0	A/A
case-111	2	74	0	0	1	A/A
case-112	1	53	1	0	0	A/A
case-113	1	67	0	1	0	G/A

case-114	2	42	0	0	0	A/A
case-115	1	67	1	1	0	A/A
case-116	2	58	0	0	0	A/A
case-117	2	48	0	0	0	G/A
case-118	1	61	1	0	0	A/A
case-119	1	64	0	0	1	G/A
case-120	1	75	1	1	0	A/A
case-121	1	63	0	0	1	G/A
case-122	1	61	0	1	1	A/A
case-123	2	56	0	0	0	A/A
case-124	1	62	1	1	1	A/A
case-125	2	68	0	0	0	A/A
case-126	1	74	0	0	1	G/A
case-127	2	70	0	0	0	A/A
case-128	1	58	1	1	0	G/A
case-129	2	54	0	0	1	A/A
case-130	1	62	0	0	0	G/G
case-131	1	74	1	0	0	G/A
case-132	2	35	0	0	0	A/A
case-133	1	63	1	1	1	A/A
case-134	2	61	0	0	0	A/A
case-135	1	53	1	1	0	A/A
case-136	1	70	0	1	0	A/A
case-137	1	48	0	1	1	G/A
case-138	2	69	0	0	0	G/A
case-139	1	65	1	0	0	A/A
case-140	2	61	0	0	0	G/G
case-141	1	61	0	0	0	A/A
case-142	2	73	0	0	1	G/A
case-143	1	52	1	0	0	A/A
case-144	1	64	0	1	0	A/A
case-145	1	60	1	0	0	A/A
case-146	1	55	1	1	1	G/A
case-147	1	70	1	0	0	A/A
case-148	1	84	1	1	1	A/A
case-149	1	64	1	0	1	A/A
case-150	2	71	0	0	0	A/A
case-151	1	67	1	0	1	G/A
case-152	1	51	1	0	0	A/A
case-153	1	53	1	0	0	A/A
case-154	1	64	1	1	0	A/A
case-155	1	54	0	0	0	A/A
case-156	1	56	1	1	0	A/A
case-157	2	63	0	0	1	A/A
case-158	1	51	1	1	0	A/A
case-159	1	53	0	1	1	A/A
case-160	2	51	0	0	0	A/A
case-161	1	63	1	1	0	A/A
case-162	1	53	1	1	0	G/G
case-163	1	56	0	0	0	A/A
case-164	1	69	1	1	0	G/A
case-165	1	76	1	1	0	A/A
case-166	1	62	1	0	0	A/A
case-167	2	61	0	0	0	A/A
case-168	1	67	0	1	0	A/A
case-169	2	64	0	0	0	A/A
case-170	1	73	0	1	0	A/A
case-171	2	82	0	0	0	A/A

case-172	2	47	0	0	0	G/A
case-173	2	48	0	0	0	A/A
case-174	1	49	1	1	0	G/A
case-175	2	40	0	0	0	A/A
case-176	2	77	0	0	0	G/A
case-177	1	54	1	0	1	G/A
case-178	1	71	0	0	0	G/A
case-179	1	76	1	1	0	A/A
case-180	1	59	1	0	0	G/G
case-181	1	72	0	0	0	A/A
case-182	2	73	0	0	1	A/A
case-183	2	30	0	0	0	A/A
case-184	1	64	1	1	0	A/A
case-185	2	81	0	0	0	G/A
case-186	1	63	1	0	1	A/A
case-187	1	69	1	1	1	G/A
case-188	1	78	1	1	0	G/A
case-189	2	78	0	0	0	A/A
case-190	1	67	1	0	0	G/A
case-191	1	53	1	1	0	A/A
case-192	1	58	1	0	0	A/A
case-193	1	69	0	0	0	A/A
case-194	1	72	1	0	0	G/A
case-195	1	70	1	1	0	A/A
case-196	2	56	0	0	0	G/A
case-197	1	69	1	1	0	G/A
case-198	1	79	0	0	0	G/A
case-199	2	57	0	0	0	A/A
case-200	1	56	0	0	0	A/A
case-201	1	53	1	1	1	G/A
case-202	1	57	1	0	0	A/A
case-203	1	62	0	0	1	A/A
case-204	1	67	0	0	0	A/A
case-205	2	71	0	0	0	A/A
case-206	1	66	1	1	0	G/A
case-207	1	46	1	1	0	A/A
case-208	2	51	0	0	0	A/A
case-209	1	75	0	0	0	G/A
case-210	2	49	0	0	1	A/A
case-211	2	61	0	0	0	A/A
case-212	1	66	1	1	0	A/A
case-213	2	82	0	0	0	G/A
case-214	1	69	1	0	1	A/A
case-215	1	58	1	0	1	G/A
case-216	1	69	1	0	0	G/A
case-217	1	71	0	0	1	G/G
case-218	1	72	0	0	0	G/A
case-219	2	78	0	0	1	A/A
case-220	2	56	0	0	0	G/A
case-221	1	59	1	1	1	G/A
case-222	1	60	0	0	0	A/A
case-223	1	50	0	0	0	A/A
case-224	1	60	1	1	0	A/A
case-225	2	55	0	0	0	A/A
case-226	1	47	1	0	1	G/A
case-227	2	47	0	0	0	A/A
case-228	1	48	1	0	0	A/A
case-229	2	33	0	0	0	G/A

case-230	1	42	1	1	0	A/A
case-231	1	79	0	0	0	A/A
case-232	1	58	1	1	0	G/A
case-233	2	63	0	0	0	A/A
case-234	1	50	0	0	0	A/A
case-235	2	33	0	0	0	A/A
case-236	1	60	0	0	1	A/A
case-237	1	70	0	0	0	G/A
case-238	1	56	1	1	0	A/A
case-239	1	61	1	1	0	A/A
case-240	1	70	1	1	0	G/A
case-241	1	40	1	0	0	G/A
case-242	2	64	0	0	0	G/A
case-243	2	71	0	0	1	A/A
case-244	1	62	0	0	1	A/A
case-245	2	35	0	0	0	A/A
case-246	2	79	0	0	0	G/A
case-247	1	70	1	0	0	A/A
case-248	1	51	1	1	0	A/A
case-249	1	60	0	0	0	A/A
case-250	1	72	0	0	0	G/A
case-251	1	58	0	0	1	G/A
case-252	1	61	0	1	0	A/A
case-253	2	65	0	0	1	A/A
case-254	1	69	1	0	0	G/A
case-255	2	74	0	0	0	A/A
case-256	2	51	0	0	0	A/A
case-257	1	76	0	0	1	A/A
case-258	1	58	1	0	0	G/A
case-259	1	68	0	0	0	?
case-260	1	58	1	0	1	A/A
case-261	1	56	0	0	0	A/A
case-262	1	52	1	1	0	A/A
case-263	2	46	0	0	0	G/G
case-264	1	69	0	0	0	G/A
case-265	1	55	1	1	0	A/A
case-266	1	51	1	0	0	A/A
case-267	1	42	0	0	1	G/A
case-268	2	51	0	0	0	A/A
case-269	1	69	0	0	1	A/A
case-270	1	57	1	1	0	A/A
case-271	1	61	0	0	0	A/A
case-272	2	46	0	0	0	A/A
case-273	1	58	1	1	0	A/A
case-274	1	56	1	0	0	A/A
case-275	1	53	1	0	0	A/A
case-276	1	64	0	0	1	A/A
case-277	1	83	0	0	0	A/A
case-278	1	68	1	0	0	A/A
case-279	1	54	1	1	0	G/A
case-280	2	45	0	0	1	G/A
case-281	1	62	1	0	0	A/A
case-282	1	64	1	0	0	G/A
case-283	1	63	0	0	1	G/G
case-284	2	46	0	0	0	A/A
case-285	1	37	0	0	0	G/A
case-286	2	38	0	0	0	A/A
case-287	1	60	1	1	1	A/A

case-288	1	64	0	0	0	G/A
case-289	1	54	1	0	0	A/A
case-290	2	40	0	0	0	A/A
case-291	2	52	0	0	0	G/A
case-292	2	57	0	0	0	A/A
case-293	1	56	1	1	0	G/A
case-294	2	62	0	0	0	A/A
case-295	1	61	0	0	0	A/A
case-296	1	52	1	0	1	A/A
case-297	1	41	1	0	0	G/G
case-298	1	68	0	0	0	A/A
case-299	2	65	0	0	1	A/A
case-300	1	50	0	0	1	A/A
case-301	1	64	1	0	1	A/A
case-302	1	56	1	1	0	A/A
case-303	2	52	0	0	0	A/A
case-304	1	68	1	0	0	A/A
case-305	1	63	0	0	0	A/A
case-306	2	48	0	0	0	G/A
case-307	2	29	0	0	0	A/A
case-308	2	66	0	0	0	A/A
case-309	1	53	1	1	1	A/A
case-310	1	64	0	0	1	A/A
case-311	1	72	1	1	0	A/A
case-312	2	63	0	0	1	A/A
case-313	2	54	0	0	1	A/A
case-314	1	45	0	0	0	A/A
case-315	1	58	0	0	0	G/A
case-316	2	27	0	0	0	A/A
case-317	1	66	0	0	0	A/A
case-318	1	49	0	0	0	A/A
case-319	1	47	1	1	0	A/A
case-320	2	51	0	0	0	A/A
case-321	2	67	0	0	0	A/A
case-322	2	44	0	0	0	A/A
case-323	1	70	1	1	0	A/A
case-324	1	72	1	0	0	G/G
case-325	1	58	1	0	0	G/A
case-326	1	49	1	0	0	A/A
case-327	1	70	0	0	1	A/A
case-328	1	64	0	1	0	A/A
case-329	2	52	0	0	1	G/A
case-330	1	68	0	1	1	A/A
case-331	1	60	1	1	0	G/G
case-332	1	52	1	0	1	A/A
case-333	2	69	0	0	1	A/A
case-334	2	67	0	0	0	A/A
case-335	1	72	1	0	1	A/A
case-336	1	69	1	0	0	A/A
case-337	1	62	0	0	1	A/A
case-338	1	66	0	1	1	A/A
case-339	2	49	1	0	0	G/A
case-340	1	71	0	0	0	A/A
case-341	1	38	1	1	1	A/A
case-342	2	54	0	0	0	A/A
case-343	2	74	0	0	0	G/A
case-344	1	71	0	0	1	A/A
case-345	1	57	0	0	0	A/A

case-346	1	59	0	0	0	G/A
case-347	1	69	1	1	0	A/A
case-348	2	52	0	0	0	A/A
case-349	2	49	0	0	0	G/A
case-350	1	34	1	0	0	A/A
case-351	1	61	1	1	1	A/A
case-352	2	66	0	0	0	G/A
case-353	1	59	0	0	0	G/A
case-354	1	73	1	1	1	A/A
case-355	1	48	0	0	1	A/A
case-356	1	67	1	1	1	A/A
case-357	1	59	0	1	0	A/A
case-358	2	76	0	0	1	A/A
case-359	1	72	0	0	1	A/A
case-360	1	64	1	1	0	A/A
case-361	1	69	1	1	0	A/A
case-362	2	65	0	0	0	A/A
case-363	1	64	0	0	0	G/G
case-364	1	70	1	0	0	A/A
case-365	1	77	1	0	0	A/A
case-366	1	63	1	1	0	G/A
case-367	1	60	1	0	0	A/A
case-368	2	43	0	0	0	G/A
case-369	1	68	0	0	0	A/A
case-370	2	71	0	0	0	G/A
case-371	1	45	1	1	0	G/A
case-372	1	56	1	1	0	A/A
case-373	2	42	0	0	0	G/A
case-374	1	59	0	0	0	G/A
case-375	1	61	1	0	1	A/A
case-376	1	45	1	1	1	G/A
case-377	1	63	0	0	0	G/A
case-378	1	62	1	0	0	A/A
case-379	2	66	0	0	1	A/A
case-380	1	61	1	0	0	A/A
case-381	1	65	1	1	0	G/G
case-382	1	59	1	1	0	A/A
case-383	1	49	0	1	1	G/A
case-384	1	61	1	1	0	A/A
case-385	2	58	0	0	0	G/A
case-386	1	64	0	1	1	G/A
case-387	1	72	0	0	0	A/A
case-388	1	75	0	0	0	A/A
case-389	1	42	1	1	0	A/A
case-390	1	61	1	0	0	A/A
case-391	1	50	1	0	0	G/A
case-392	1	63	0	0	0	A/A
case-393	1	83	0	0	0	G/A
case-394	1	72	0	0	0	A/A
case-395	2	62	0	0	1	G/A
case-396	2	70	0	0	0	A/A
case-397	1	53	0	0	0	A/A
case-398	2	49	0	0	1	A/A
case-399	2	65	0	0	1	A/A
case-400	1	71	1	0	1	A/A
case-401	1	59	1	0	0	G/G
case-402	1	52	0	0	0	A/A
case-403	1	79	0	0	0	G/G

case-404	2	55	0	0	1	A/A
case-405	2	53	0	0	0	A/A
case-406	1	70	1	0	1	G/A
case-407	1	71	0	0	0	A/A
case-408	1	52	1	0	0	G/A
case-409	1	66	0	1	0	A/A
case-410	2	55	0	0	0	A/A
case-411	2	66	0	0	1	A/A
case-412	2	63	0	0	1	A/A
case-413	2	49	0	0	0	A/A
case-414	1	71	0	0	0	A/A
case-415	1	63	0	0	0	A/A
case-416	1	64	0	0	0	G/A
case-417	1	63	0	1	1	A/A
case-418	1	51	1	0	0	A/A
case-419	2	47	0	0	0	A/A
case-420	1	49	0	0	0	A/A
case-421	1	43	0	0	1	A/A
case-422	2	73	0	0	0	A/A
case-423	1	61	0	0	0	A/A
case-424	1	54	1	1	0	A/A
case-425	1	64	1	1	0	A/A
case-426	1	58	1	1	1	?
case-427	1	77	0	1	0	A/A
case-428	2	69	0	0	0	A/A
case-429	1	67	0	0	0	A/A
case-430	1	79	0	0	1	A/A
case-431	1	60	0	0	0	G/A
case-432	1	65	1	0	0	A/A
case-433	1	54	0	0	0	A/A
case-434	1	57	0	0	1	A/A
case-435	2	57	0	0	0	G/A
case-436	1	44	0	0	1	A/A
case-437	1	70	0	0	1	A/A
case-438	1	58	0	1	0	A/A
case-439	2	43	0	0	0	A/A
case-440	1	74	0	0	0	G/G
case-441	1	73	1	0	1	A/A
case-442	2	75	0	0	1	G/A
case-443	2	44	0	0	0	A/A
case-444	1	75	0	0	1	G/A
case-445	1	85	0	0	0	A/A
case-446	2	46	0	0	0	A/A
case-447	1	59	1	0	0	A/A
case-448	2	53	0	0	1	A/A
case-449	1	72	1	1	0	A/A
case-450	2	70	0	0	0	G/A
case-451	1	69	0	0	0	A/A
case-452	1	78	1	1	1	G/A
case-453	1	64	0	0	0	G/A
case-454	1	71	0	0	1	G/A
case-455	2	78	0	0	0	A/A
case-456	2	75	0	0	1	G/A
case-457	1	77	0	0	0	A/A
case-458	1	51	0	0	1	A/A
case-459	1	74	0	1	0	A/A
case-460	2	40	0	0	0	A/A
case-461	2	66	0	0	0	A/A

case-462	1	66	0	0	0	A/A
case-463	1	69	0	0	0	G/A
case-464	1	75	0	0	1	A/A
case-465	2	65	0	0	1	A/A
case-466	2	65	0	0	1	A/A
case-467	1	64	0	0	1	A/A
case-468	1	80	1	0	0	G/A
case-469	1	74	0	0	1	G/G
case-470	2	50	0	0	1	A/A
case-471	2	66	0	0	0	A/A
case-472	2	47	0	0	1	G/A
case-473	1	69	1	0	0	A/A
case-474	1	60	1	0	0	A/A
case-475	1	55	0	0	0	A/A
case-476	2	67	0	0	0	A/A
case-477	1	49	0	0	1	G/A
case-478	1	65	1	0	1	A/A
case-479	1	62	0	0	0	G/A
case-480	2	78	0	0	0	A/A
case-481	1	63	0	0	1	A/A
case-482	1	61	1	0	1	A/A
case-483	2	88	0	1	0	A/A
case-484	1	83	0	0	0	A/A
case-485	2	72	0	0	0	A/A
case-486	2	73	0	0	0	A/A
case-487	2	61	0	0	0	A/A
case-488	1	69	1	0	1	A/A
case-489	1	73	1	0	0	A/A
case-490	2	55	0	0	0	G/A
control-001	1	55	0	0	1	A/A
control-002	2	53	0	0	1	A/A
control-003	1	59	0	0	1	A/A
control-004	1	74	0	0	1	A/A
control-005	2	54	0	0	0	G/A
control-006	2	70	0	0	1	G/A
control-007	1	65	1	0	0	G/A
control-008	1	62	0	0	0	A/A
control-009	2	77	0	0	1	A/A
control-010	2	56	0	0	0	A/A
control-011	1	63	0	0	0	A/A
control-012	1	51	0	0	1	G/A
control-013	2	61	0	0	0	A/A
control-014	1	57	1	0	1	A/A
control-015	1	81	0	0	1	A/A
control-016	1	79	0	0	1	A/A
control-017	1	53	1	0	0	G/A
control-018	1	65	0	0	0	A/A
control-019	1	56	0	1	1	A/A
control-020	1	64	1	1	1	A/A
control-021	1	62	0	0	1	A/A
control-022	1	72	1	0	0	A/A
control-023	1	66	0	0	1	A/A
control-024	1	29	1	0	1	G/A
control-025	2	71	0	0	0	A/A
control-026	1	38	0	0	1	A/A
control-027	1	71	0	0	1	G/A
control-028	1	60	1	0	1	A/A
control-029	2	53	0	0	1	A/A

control-030	1	58	0	0	0	A/A
control-031	2	52	0	0	0	A/A
control-032	1	67	1	0	0	A/A
control-033	2	69	0	0	1	A/A
control-034	1	80	0	0	1	G/A
control-035	1	77	1	0	1	A/A
control-036	2	38	0	0	0	A/A
control-037	1	28	0	0	0	G/A
control-038	2	65	0	0	1	A/A
control-039	2	52	0	0	0	A/A
control-040	1	66	0	0	0	A/A
control-041	2	46	0	0	0	G/A
control-042	1	56	1	0	1	A/A
control-043	2	68	0	0	1	A/A
control-044	1	59	0	0	0	A/A
control-045	1	62	0	0	1	A/A
control-046	1	71	0	0	0	A/A
control-047	1	60	1	1	0	A/A
control-048	1	56	1	0	0	G/A
control-049	1	73	1	0	0	A/A
control-050	1	60	0	0	0	A/A
control-051	2	72	0	0	0	A/A
control-052	2	61	0	0	0	A/A
control-053	2	66	0	0	0	A/A
control-054	1	61	1	0	0	A/A
control-055	1	71	1	0	0	G/A
control-056	1	77	0	0	0	A/A
control-057	1	64	1	0	0	A/A
control-058	2	54	0	0	0	G/A
control-059	1	63	0	0	0	A/A
control-060	2	56	0	0	0	A/A
control-061	1	63	1	0	0	G/G
control-062	2	71	0	0	1	A/A
control-063	1	72	1	0	0	A/A
control-064	1	46	0	0	0	A/A
control-065	1	62	1	0	0	A/A
control-066	2	59	0	0	0	A/A
control-067	2	79	0	0	0	A/A
control-068	2	60	0	0	1	A/A
control-069	2	52	0	0	1	A/A
control-070	1	56	1	0	0	A/A
control-071	1	59	1	0	0	G/A
control-072	1	61	0	0	1	A/A
control-073	1	63	0	0	1	A/A
control-074	1	75	1	0	1	A/A
control-075	1	72	0	0	0	G/A
control-076	1	64	0	1	0	A/A
control-077	2	67	0	0	1	A/A
control-078	1	58	1	0	0	A/A
control-079	1	70	1	1	1	A/A
control-080	1	58	0	0	0	A/A
control-081	1	78	0	0	1	G/A
control-082	2	61	0	0	0	A/A
control-083	1	67	0	0	1	G/A
control-084	1	41	1	0	1	A/A
control-085	2	69	0	0	0	A/A
control-086	1	62	0	0	0	A/A
control-087	1	69	0	0	0	A/A

control-088	1	81	0	0	0	A/A
control-089	1	57	1	0	0	G/A
control-090	2	51	0	0	1	A/A
control-091	1	55	1	0	0	A/A
control-092	2	37	0	0	0	A/A
control-093	1	65	1	1	0	A/A
control-094	1	57	0	0	1	G/A
control-095	1	80	0	0	0	A/A
control-096	2	49	0	0	0	A/A
control-097	2	46	0	0	0	A/A
control-098	1	65	0	0	0	A/A
control-099	2	64	0	0	1	G/A
control-100	2	65	0	0	0	A/A
control-101	1	69	1	0	0	A/A
control-102	1	78	0	0	1	A/A
control-103	1	43	0	0	0	A/A
control-104	1	64	0	0	0	G/A
control-105	2	56	0	0	1	G/A
control-106	2	78	0	0	1	A/A
control-107	2	52	0	0	1	A/A
control-108	2	57	0	0	0	A/A
control-109	2	80	0	0	1	A/A
control-110	2	73	0	0	0	G/A
control-111	2	67	0	0	0	G/A
control-112	2	53	0	0	0	A/A
control-113	1	48	1	0	1	A/A
control-114	1	57	1	0	0	A/A
control-115	1	62	1	0	1	A/A
control-116	2	71	0	0	0	A/A
control-117	1	69	1	1	0	G/A
control-118	2	53	0	0	0	A/A
control-119	1	75	0	0	1	A/A
control-120	2	49	0	0	1	G/A
control-121	2	61	0	0	0	A/A
control-122	2	62	0	0	1	A/A
control-123	1	53	1	0	1	A/A
control-124	1	62	0	0	1	G/A
control-125	2	78	0	0	1	A/A
control-126	1	63	0	0	0	A/A
control-127	1	33	0	0	0	A/A
control-128	1	81	0	0	1	A/A
control-129	2	59	0	0	1	A/A
control-130	2	70	0	0	0	G/A
control-131	2	56	0	0	0	A/A
control-132	1	69	0	0	1	A/A
control-133	1	58	0	0	0	A/A
control-134	1	60	1	0	0	A/A
control-135	1	59	0	0	1	A/A
control-136	1	76	1	0	0	G/G
control-137	1	52	0	0	0	A/A
control-138	2	34	0	0	0	A/A
control-139	1	60	1	0	1	A/A
control-140	1	76	1	0	0	A/A
control-141	1	81	1	0	0	A/A
control-142	2	50	0	0	1	G/A
control-143	1	57	0	0	0	A/A
control-144	1	51	0	0	0	G/G
control-145	1	76	0	0	0	A/A

control-146	1	64	0	0	0	A/A
control-147	1	60	0	0	0	A/A
control-148	2	65	0	0	1	A/A
control-149	1	55	0	0	1	G/A
control-150	2	70	0	0	0	A/A
control-151	1	38	1	0	0	A/A
control-152	1	64	0	0	1	A/A
control-153	1	62	0	0	0	A/A
control-154	1	54	1	0	0	G/A
control-155	1	64	0	0	0	A/A
control-156	1	56	0	0	0	A/A
control-157	2	60	0	0	1	A/A
control-158	1	64	0	0	0	A/A
control-159	1	78	0	0	1	G/A
control-160	1	58	0	0	0	A/A
control-161	1	46	0	0	0	A/A
control-162	2	72	0	0	0	G/A
control-163	1	44	1	0	1	A/A
control-164	2	41	0	0	0	A/A
control-165	1	46	1	0	1	G/A
control-166	1	82	1	0	0	A/A
control-167	2	67	0	0	0	G/A
control-168	2	65	0	0	0	G/A
control-169	2	66	0	0	1	G/A
control-170	1	61	1	0	0	A/A
control-171	1	52	0	0	0	A/A
control-172	2	67	0	0	1	A/A
control-173	1	60	1	0	1	A/A
control-174	1	56	1	0	0	A/A
control-175	1	60	0	0	1	G/A
control-176	1	57	0	0	1	A/A
control-177	2	65	0	0	0	A/A
control-178	2	70	0	0	0	G/G
control-179	2	69	0	0	1	G/A
control-180	1	74	0	0	0	G/A
control-181	1	63	1	0	1	A/A
control-182	1	53	1	0	0	A/A
control-183	2	25	0	0	0	A/A
control-184	2	79	0	0	0	A/A
control-185	2	43	0	0	0	A/A
control-186	1	62	1	0	1	G/A
control-187	2	74	0	0	1	G/A
control-188	2	73	0	0	0	G/G
control-189	2	62	0	0	0	A/A
control-190	1	74	1	0	0	G/A
control-191	1	58	0	0	0	A/A
control-192	2	69	0	0	1	A/A
control-193	2	51	0	0	0	A/A
control-194	1	61	0	0	1	A/A
control-195	1	66	0	0	1	A/A
control-196	1	74	1	0	0	A/A
control-197	1	55	0	0	1	A/A
control-198	2	72	0	0	0	A/A
control-199	1	56	0	0	0	A/A
control-200	2	78	0	0	1	G/A
control-201	1	43	0	0	1	A/A
control-202	2	63	0	0	1	G/A
control-203	2	61	0	0	1	A/A

control-204	1	59	1	0	1	A/A
control-205	1	60	1	0	0	A/A
control-206	1	77	0	0	1	A/A
control-207	1	79	0	0	1	G/A
control-208	2	63	0	0	1	A/A
control-209	2	39	0	0	1	A/A
control-210	1	42	0	0	1	A/A
control-211	1	68	0	1	0	A/A
control-212	1	58	0	0	0	A/A
control-213	1	54	0	0	0	A/A
control-214	1	57	1	0	1	A/A
control-215	1	66	0	0	0	G/A
control-216	1	74	0	0	1	A/A
control-217	2	50	0	0	0	A/A
control-218	1	68	1	0	1	A/A
control-219	1	60	0	0	1	G/A
control-220	1	44	1	0	1	G/A
control-221	2	59	0	0	1	G/A
control-222	1	73	0	0	1	A/A
control-223	1	75	1	0	1	A/A
control-224	1	65	0	0	0	G/A
control-225	1	62	1	0	0	A/A
control-226	1	57	0	0	0	A/A
control-227	2	76	0	0	1	A/A
control-228	1	46	0	0	1	A/A
control-229	1	47	1	0	1	A/A
control-230	2	60	0	0	1	A/A
control-231	1	59	0	0	0	A/A
control-232	2	52	0	0	0	A/A
control-233	1	52	0	0	1	A/A
control-234	1	76	0	0	1	A/A
control-235	1	75	0	0	1	A/A
control-236	2	51	0	0	1	A/A
control-237	1	66	0	0	1	A/A
control-238	1	47	1	0	1	G/A
control-239	2	61	0	0	0	A/A
control-240	1	44	0	0	0	A/A
control-241	2	62	0	0	1	A/A
control-242	1	40	0	0	1	A/A
control-243	1	50	0	1	1	A/A
control-244	2	59	0	0	0	G/A
control-245	1	48	0	0	1	A/A
control-246	1	74	1	1	1	A/A
control-247	1	57	0	0	0	G/A
control-248	1	69	1	0	1	A/A
control-249	1	62	0	0	1	A/A
control-250	2	57	0	0	1	A/A
control-251	1	37	0	0	0	A/A
control-252	1	64	0	0	1	A/A
control-253	1	64	0	0	0	A/A
control-254	1	45	0	0	1	A/A
control-255	1	77	0	0	0	G/A
control-256	1	60	0	0	0	A/A
control-257	1	68	0	0	0	G/A
control-258	1	49	1	1	0	G/A
control-259	1	49	0	0	0	G/A
control-260	1	43	0	0	1	A/A
control-261	1	81	0	0	1	G/A

control-262	1	46	0	0	1	A/A
control-263	2	55	0	0	1	A/A
control-264	1	79	1	1	0	G/A
control-265	1	71	1	1	0	A/A
control-266	1	64	0	0	0	A/A
control-267	2	57	0	0	0	G/A
control-268	2	78	0	0	1	G/A
control-269	1	58	1	0	1	A/A
control-270	1	74	0	0	0	A/A
control-271	1	41	0	0	1	A/A
control-272	2	54	0	0	1	A/A
control-273	1	59	1	1	0	A/A
control-274	1	70	0	0	0	A/A
control-275	1	55	1	0	1	G/A
control-276	1	35	0	0	0	G/A
control-277	1	58	0	0	0	A/A
control-278	1	59	0	0	1	A/A
control-279	1	60	0	0	0	G/A
control-280	1	58	0	0	0	A/A
control-281	2	60	0	0	0	A/A
control-282	2	61	0	0	0	A/A
control-283	2	67	0	0	0	G/A
control-284	1	59	1	0	1	A/A
control-285	2	55	0	0	0	A/A
control-286	1	66	0	0	0	A/A
control-287	1	64	0	0	1	A/A
control-288	1	71	0	0	0	A/A
control-289	1	56	1	0	1	A/A
control-290	2	61	0	0	1	A/A
control-291	1	73	1	0	0	A/A
control-292	1	60	1	0	0	G/A
control-293	2	66	0	0	0	G/A
control-294	2	63	0	0	1	G/A
control-295	1	65	1	0	1	A/A
control-296	1	60	0	0	0	G/A
control-297	2	51	0	0	0	A/A
control-298	1	57	0	0	1	A/A
control-299	2	79	0	0	1	G/A
control-300	1	55	1	1	0	A/A
control-301	1	51	0	0	1	A/A
control-302	2	66	0	0	0	A/A
control-303	1	53	1	0	0	A/A
control-304	1	61	0	0	1	G/G
control-305	2	79	0	0	0	G/A
control-306	1	62	0	0	1	A/A
control-307	1	60	1	0	1	G/A
control-308	1	71	1	1	1	G/A
control-309	1	67	0	0	0	A/A
control-310	2	69	0	0	0	A/A
control-311	1	62	0	0	0	A/A
control-312	1	61	1	0	0	G/A
control-313	1	62	1	0	0	A/A
control-314	2	70	0	0	1	A/A
control-315	2	56	0	0	1	A/A
control-316	2	68	0	0	1	G/A
control-317	2	53	0	0	1	A/A
control-318	2	56	0	0	1	A/A
control-319	2	48	0	0	0	A/A

control-320	1	43	1	0	0	A/A
control-321	1	61	0	0	1	G/A
control-322	1	67	0	0	1	A/A
control-323	1	57	0	0	0	A/A
control-324	1	39	0	0	1	A/A
control-325	1	63	0	0	0	A/A
control-326	1	74	1	0	0	G/A
control-327	1	76	0	0	1	A/A
control-328	1	67	0	0	0	A/A
control-329	1	61	0	0	0	A/A
control-330	1	74	0	1	0	G/A
control-331	2	65	0	0	1	A/A
control-332	1	71	1	0	0	A/A
control-333	1	59	1	0	0	G/A
control-334	1	69	1	0	0	A/A
control-335	2	78	0	0	0	A/A
control-336	1	59	0	0	1	A/A
control-337	1	63	1	0	1	G/A
control-338	1	82	1	0	0	G/A
control-339	2	63	0	0	1	G/A
control-340	1	71	1	0	1	G/A
control-341	1	64	0	1	0	A/A
control-342	1	56	0	0	0	G/A
control-343	2	72	0	0	1	A/A
control-344	1	62	1	0	0	A/A
control-345	2	51	0	0	0	A/A
control-346	1	58	1	0	0	G/A
control-347	1	60	0	0	0	G/A
control-348	2	66	0	0	0	G/A
control-349	2	70	0	0	0	G/A
control-350	2	81	0	0	1	G/A
control-351	1	57	0	0	1	A/A
control-352	2	61	0	0	0	G/A
control-353	2	59	0	0	0	A/A
control-354	1	79	0	0	0	A/A
control-355	2	54	0	0	0	A/A
control-356	1	64	0	0	0	A/A
control-357	2	48	0	0	1	A/A
control-358	1	74	1	0	1	A/A
control-359	1	63	1	0	0	A/A
control-360	2	69	0	0	0	A/A
control-361	2	74	0	0	1	G/A
control-362	1	59	0	0	1	A/A
control-363	2	63	0	0	1	A/A
control-364	1	73	0	0	1	G/A
control-365	2	68	0	0	0	A/A
control-366	1	76	0	0	0	A/A
control-367	1	65	0	0	0	G/A
control-368	1	73	0	0	1	G/G
control-369	2	59	0	0	0	A/A
control-370	1	65	1	0	0	A/A
control-371	1	66	0	0	0	G/A
control-372	2	67	0	0	0	A/A
control-373	2	55	0	0	0	A/A
control-374	1	78	0	0	1	G/A
control-375	1	71	0	0	1	G/A
control-376	2	67	0	0	0	A/A
control-377	1	53	0	0	1	A/A

control-378	2	67	0	0	1	A/A
control-379	2	68	0	0	1	G/A
control-380	2	82	0	0	0	G/A
control-381	1	55	1	0	0	A/A
control-382	2	63	0	0	1	A/A
control-383	1	38	0	0	1	A/A
control-384	2	72	0	0	0	A/A
control-385	1	56	1	0	1	A/A
control-386	1	80	0	0	0	A/A
control-387	1	56	0	0	1	A/A
control-388	1	65	0	0	0	A/A
control-389	1	59	0	0	1	A/A
control-390	1	72	0	0	0	A/A
control-391	1	59	1	0	1	A/A
control-392	1	77	1	0	0	A/A
control-393	1	60	0	0	0	G/A
control-394	1	56	1	0	0	G/A
control-395	1	49	0	0	0	A/A
control-396	1	62	0	0	0	A/A
control-397	2	68	0	0	0	A/A
control-398	1	70	1	0	1	G/A
control-399	2	65	0	0	1	A/A
control-400	2	77	0	0	1	A/A
control-401	1	49	1	0	0	A/A
control-402	1	71	1	0	1	A/A
control-403	1	52	0	0	0	A/A
control-404	1	47	0	0	0	G/A
control-405	1	48	0	0	1	A/A
control-406	2	46	0	0	0	A/A
control-407	2	51	0	0	0	G/A
control-408	1	67	1	0	1	A/A
control-409	1	78	0	0	0	A/A
control-410	1	48	1	0	0	G/A
control-411	2	56	0	0	0	A/A
control-412	2	51	0	0	1	A/A
control-413	1	82	0	0	1	G/A
control-414	2	63	0	0	1	A/A
control-415	2	54	0	0	1	A/A
control-416	2	52	0	0	1	A/A
control-417	1	52	0	0	1	G/A
control-418	1	58	1	0	0	A/A
control-419	1	78	0	0	0	A/A
control-420	1	51	0	0	0	A/A
control-421	2	68	0	0	1	G/A
control-422	1	52	0	0	0	A/A
control-423	2	51	0	0	1	A/A
control-424	2	66	0	0	1	A/A
control-425	2	62	0	0	0	A/A
control-426	1	66	0	0	0	G/A
control-427	1	65	0	0	1	A/A
control-428	2	60	0	0	0	A/A
control-429	1	48	0	0	1	A/A
control-430	2	53	0	0	1	A/A
control-431	1	70	1	0	1	A/A
control-432	2	60	0	0	1	A/A
control-433	2	63	0	0	0	A/A
control-434	2	61	0	0	1	A/A
control-435	1	78	1	0	0	G/A

control-436	1	55	0	0	0	A/A
control-437	1	69	0	0	1	A/A
control-438	1	67	0	0	1	G/A
control-439	1	62	0	0	0	A/A
control-440	2	49	0	0	0	G/A
control-441	1	55	0	0	1	A/A
control-442	1	62	1	0	0	A/A
control-443	1	48	1	0	1	A/A
control-444	1	49	1	0	1	G/A
control-445	1	61	0	0	1	A/A
control-446	2	77	0	0	1	G/A
control-447	2	53	0	0	0	A/A
control-448	1	69	1	0	0	A/A
control-449	2	70	0	0	1	A/A
control-450	1	52	0	0	0	A/A
control-451	2	61	0	0	0	A/A
control-452	2	67	0	0	0	A/A
control-453	1	30	0	0	0	G/G
control-454	1	58	0	0	0	G/A
control-455	2	61	0	0	1	A/A
control-456	1	75	0	0	0	A/A
control-457	1	54	0	0	0	A/A
control-458	2	42	0	0	0	A/A
control-459	2	61	0	0	0	G/A
control-460	1	57	0	0	0	A/A
control-461	1	50	1	0	1	A/A
control-462	2	66	0	0	0	A/A
control-463	1	55	1	0	1	A/A
control-464	2	52	0	0	1	A/A
control-465	1	41	0	0	0	A/A
control-466	2	63	0	0	1	A/A
control-467	1	59	0	0	1	A/A
control-468	1	74	1	1	0	A/A
control-469	1	68	0	0	0	G/A
control-470	1	63	1	1	1	G/A
control-471	1	60	1	0	0	G/A
control-472	1	59	0	0	1	G/A
control-473	2	57	0	0	1	A/A
control-474	1	60	1	0	1	G/A
control-475	2	42	0	0	0	A/A
control-476	1	54	1	0	0	A/A
control-477	2	42	0	0	1	A/A
control-478	1	51	0	0	0	A/A
control-479	1	50	0	0	1	A/A
control-480	1	58	0	0	1	G/A
control-481	2	65	1	0	1	G/A
control-482	2	73	0	0	0	G/G
control-483	1	51	0	0	0	A/A
control-484	1	64	0	0	1	A/A
control-485	1	27	0	0	0	G/A
control-486	1	63	1	0	0	A/A
control-487	1	53	1	0	0	A/A
control-488	2	64	0	0	0	A/A
control-489	1	55	1	0	0	A/A
control-490	1	39	1	0	0	G/A
control-491	1	69	0	0	1	A/A
control-492	1	50	1	0	0	A/A
control-493	1	76	1	0	0	A/A

control-494	1	79	1	0	0	A/A
control-495	1	52	1	0	0	A/A
control-496	2	79	0	0	1	A/A
control-497	2	58	0	0	1	A/A
control-498	1	43	0	0	0	A/A
control-499	2	53	0	0	0	A/A
control-500	2	71	0	0	1	G/A
control-501	1	78	0	0	0	G/A
control-502	1	63	1	0	0	G/A
control-503	1	57	1	0	1	G/A
control-504	2	60	0	0	0	A/A
control-505	1	67	0	0	1	G/A
control-506	1	66	0	0	1	A/A
control-507	1	62	0	0	1	G/A
control-508	2	61	0	0	1	A/A
control-509	1	61	1	0	1	A/A
control-510	1	66	0	0	1	A/A
control-511	1	52	0	0	1	G/A
control-512	2	71	0	0	1	A/A
control-513	2	73	0	0	1	G/A
control-514	1	60	1	0	0	G/A
control-515	1	72	1	1	0	A/A
control-516	1	70	0	0	0	A/A
control-517	1	68	0	0	0	A/A
control-518	1	71	1	0	0	A/A
control-519	2	69	0	0	1	A/A
control-520	2	61	0	0	1	G/A
control-521	1	69	0	0	1	A/A
control-522	1	74	1	0	1	A/A
control-523	2	52	0	0	1	A/A
control-524	1	41	0	0	0	A/A
control-525	1	52	0	0	0	A/A
control-526	2	44	0	0	0	G/G
control-527	2	50	0	0	0	A/A
control-528	1	63	0	0	1	G/A
control-529	1	59	1	0	1	A/A
control-530	1	44	0	0	1	G/A
control-531	2	66	0	0	0	G/A
control-532	1	59	1	1	0	A/A
control-533	1	61	1	0	1	G/A
control-534	2	53	0	0	1	A/A
control-535	1	66	1	0	1	A/A
control-536	2	54	0	0	0	A/A
control-537	2	51	0	0	1	G/A
control-538	2	61	0	0	0	A/A
control-539	1	56	0	0	1	A/A
control-540	1	52	0	0	1	G/A
control-541	2	49	0	0	1	A/A
control-542	2	48	0	0	0	A/A
control-543	2	45	0	0	1	A/A
control-544	2	47	0	0	0	G/A
control-545	2	65	0	0	1	A/A
control-546	1	73	1	0	0	A/A
control-547	2	59	0	0	0	A/A
control-548	1	59	0	0	0	G/A
control-549	1	79	0	0	1	A/A
control-550	1	67	0	0	0	A/A
control-551	1	78	0	0	0	G/A

control-552	1	59	0	0	1	G/A
control-553	1	71	0	0	0	A/A
control-554	1	59	0	0	1	A/A
control-555	1	47	1	0	1	A/A
control-556	2	68	0	0	0	A/A
control-557	1	64	0	1	0	A/A
control-558	2	50	0	0	1	G/A
control-559	1	74	1	0	0	A/A
control-560	2	50	0	0	0	A/A
control-561	1	70	0	0	0	A/A
control-562	2	73	0	0	1	A/A
control-563	1	82	0	0	0	G/A
control-564	2	63	0	0	0	A/A
control-565	1	53	0	0	0	A/A
control-566	2	59	0	0	0	G/A
control-567	1	58	0	0	0	G/A
control-568	1	68	0	0	0	A/A
control-569	2	53	0	0	1	A/A
control-570	2	65	0	0	1	G/A
control-571	2	73	0	0	1	A/A
control-572	1	73	1	0	0	A/A
control-573	1	77	1	0	1	A/A
control-574	2	56	0	0	1	A/A
control-575	1	59	0	0	0	A/A
control-576	2	51	0	0	1	A/A
control-577	1	62	0	0	1	G/A
control-578	1	55	0	0	0	A/A
control-579	2	59	0	0	1	A/A
control-580	1	62	0	0	1	A/A
control-581	1	69	0	0	0	G/A
control-582	1	74	0	0	0	G/A
control-583	1	29	0	0	1	A/A
control-584	1	59	0	0	0	A/A
control-585	1	70	0	0	1	A/A
control-586	1	52	1	0	0	A/A
control-587	1	60	0	0	1	A/A
control-588	2	61	0	0	0	A/A
control-589	1	69	1	0	1	A/A
control-590	2	77	0	0	1	A/A
control-591	2	72	0	0	0	A/A
control-592	2	77	0	0	0	A/A
control-593	2	69	0	0	1	A/A
control-594	1	59	0	0	1	A/A
control-595	2	63	0	0	1	A/A
control-596	2	66	0	0	0	G/A
control-597	1	63	0	0	1	A/A
control-598	1	76	0	0	0	G/A
control-599	1	74	0	0	1	A/A
control-600	2	59	0	0	1	A/A
control-601	1	73	1	0	1	A/A
control-602	1	57	0	0	1	A/A
control-603	2	72	0	0	1	A/A
control-604	1	63	0	0	0	A/A
control-605	2	68	0	0	1	A/A
control-606	2	79	0	0	1	G/A
control-607	1	76	0	0	1	G/A
control-608	1	66	0	0	1	G/A
control-609	1	61	1	0	1	A/A

control-610	1	53	0	0	1	G/A
control-611	2	58	0	0	0	A/A
control-612	2	78	0	0	1	A/A
control-613	1	62	0	0	1	A/A
control-614	2	66	0	0	1	A/A
control-615	1	69	1	0	0	G/A
control-616	1	73	0	0	0	G/A
control-617	1	76	1	0	0	G/A
control-618	1	57	1	0	0	A/A
control-619	1	57	1	0	1	G/A
control-620	1	46	1	1	1	G/A
control-621	2	73	0	0	1	G/A
control-622	1	60	1	1	0	A/A
control-623	2	65	0	0	0	G/A
control-624	1	66	1	0	0	A/A
control-625	1	62	0	0	0	A/A
control-626	2	71	0	0	0	G/A
control-627	2	74	0	0	0	A/A
control-628	2	74	0	0	1	G/A
control-629	2	71	0	0	0	A/A
control-630	1	71	1	0	1	A/A
control-631	1	54	1	0	0	G/A
control-632	1	78	0	0	0	A/A
control-633	1	53	0	0	0	A/A
control-634	2	64	0	0	1	A/A
control-635	2	71	0	0	1	A/A
control-636	2	68	0	0	1	A/A
control-637	1	64	1	1	1	A/A
control-638	1	55	0	0	1	A/A
control-639	2	60	0	0	0	A/A
control-640	1	73	1	0	1	A/A
control-641	1	68	0	0	0	A/A
control-642	1	75	1	0	0	A/A
control-643	1	79	0	0	0	A/A
control-644	1	59	1	0	0	A/A
control-645	1	76	0	0	1	A/A
control-646	1	59	1	0	1	G/A
control-647	1	63	1	0	0	A/A
control-648	1	62	1	1	1	A/A
control-649	1	66	1	0	1	A/A
control-650	1	53	0	0	1	A/A
control-651	1	52	0	0	0	A/A
control-652	2	77	0	0	0	A/A
control-653	1	81	0	0	0	A/A
control-654	1	67	0	0	0	G/A
control-655	1	54	1	0	1	A/A
control-656	1	55	0	0	0	A/A
control-657	2	69	0	0	1	G/A
control-658	2	74	0	0	0	A/A
control-659	2	76	0	0	1	G/A
control-660	1	65	0	1	0	A/A
control-661	1	58	1	1	1	G/A
control-662	1	68	0	0	1	A/A
control-663	1	51	1	0	1	A/A
control-664	1	48	1	0	1	G/A
control-665	2	54	0	0	0	A/A
control-666	1	79	0	1	0	A/A
control-667	2	60	0	0	1	G/A

control-668	2	64	0	0	0	A/A
control-669	1	66	0	0	1	A/A
control-670	1	68	1	0	0	G/A
control-671	1	59	1	1	1	G/A
control-672	1	67	1	0	0	G/A
control-673	2	59	0	0	0	A/A
control-674	1	73	0	0	0	A/A
control-675	1	56	1	0	0	A/A
control-676	1	60	0	0	0	A/A
control-677	1	51	0	0	0	A/A
control-678	1	58	1	0	0	A/A
control-679	1	78	0	0	1	A/A
control-680	1	65	0	0	0	A/A
control-681	2	71	0	0	0	A/A
control-682	1	63	0	0	0	A/A
control-683	1	66	0	0	1	A/A
control-684	1	78	1	0	0	A/A
control-685	2	58	0	0	0	A/A
control-686	1	68	0	0	1	A/A
control-687	2	61	0	0	0	A/A
control-688	1	68	0	0	0	A/A
control-689	1	62	1	1	1	G/A
control-690	1	69	0	0	0	A/A
control-691	1	54	0	0	0	G/A
control-692	1	58	0	0	1	A/A
control-693	2	60	0	0	1	A/A
control-694	1	69	1	1	0	G/A
control-695	1	59	1	0	1	A/A
control-696	2	59	0	0	1	A/A
control-697	1	64	0	0	1	A/A
control-698	1	58	1	0	0	A/A
control-699	1	60	0	0	1	G/A
control-700	1	60	1	1	1	A/A
control-701	2	50	0	0	0	A/A
control-702	1	54	1	1	1	G/A
control-703	1	58	0	0	1	A/A
control-704	1	68	1	0	1	A/A
control-705	1	57	0	0	0	G/A
control-706	2	71	0	0	0	A/A
control-707	1	62	0	0	1	G/A
control-708	1	66	0	0	0	A/A
control-709	1	65	1	0	1	A/A
control-710	1	61	0	0	1	G/A
control-711	2	61	0	0	0	A/A
control-712	2	57	0	0	0	G/A
control-713	1	56	1	1	1	A/A
control-714	2	71	0	0	1	A/A
control-715	2	59	0	0	1	A/A
control-716	2	61	0	0	1	A/A
control-717	2	54	0	1	0	A/A
control-718	1	65	0	0	1	A/A
control-719	1	64	1	0	0	G/A
control-720	2	66	0	0	1	A/A
control-721	1	56	1	1	0	A/A
control-722	1	51	1	0	1	A/A
control-723	1	54	0	0	1	G/A
control-724	1	75	1	0	1	G/A
control-725	1	59	1	1	0	G/A

control-726	1	57	1	0	1	A/A
control-727	1	66	0	1	1	G/A
control-728	1	73	0	0	1	A/A
control-729	1	63	0	1	0	G/A
control-730	2	77	0	0	0	G/A
control-731	1	52	1	1	0	A/A
control-732	2	65	0	0	0	A/A
control-733	1	59	1	0	0	A/A
control-734	1	67	0	0	0	G/G
control-735	1	64	0	1	1	A/A
control-736	1	57	1	0	1	A/A
control-737	1	52	0	0	1	A/A
control-738	1	73	0	0	0	A/A
control-739	1	51	0	0	1	A/A
control-740	1	68	1	1	1	G/A
control-741	1	63	0	0	0	A/A
control-742	2	70	0	0	0	A/A
control-743	1	63	1	0	1	G/A
control-744	2	68	0	0	1	A/A
control-745	2	55	0	0	1	A/A
control-746	1	55	1	1	1	A/A
control-747	1	68	1	1	0	G/A
control-748	1	64	1	1	0	G/A
control-749	1	60	0	0	0	G/A
control-750	1	73	1	1	1	A/A
control-751	1	50	0	0	0	A/A
control-752	1	51	0	1	1	G/A
control-753	1	77	0	0	1	A/A
control-754	1	58	1	1	0	A/A
control-755	1	57	0	0	0	G/G
control-756	1	61	1	1	1	G/A
control-757	1	63	1	0	0	G/A
control-758	2	70	0	0	1	A/A
control-759	1	70	1	0	1	A/A
control-760	1	60	1	0	0	A/A
control-761	1	79	0	0	0	A/A
control-762	2	66	0	0	1	A/A
control-763	1	70	1	1	1	A/A
control-764	1	65	1	0	0	G/A
control-765	2	63	0	0	0	G/A
control-766	2	59	0	0	1	A/A
control-767	1	59	1	1	0	G/A
control-768	1	61	1	1	0	A/A
control-769	1	50	0	1	1	G/A
control-770	1	71	1	0	0	A/A
control-771	2	50	0	0	1	A/A
control-772	1	65	0	0	1	A/A
control-773	1	69	0	0	1	A/A
control-774	2	73	0	0	1	A/A
control-775	1	68	0	0	0	A/A
control-776	2	56	0	0	0	G/A
control-777	1	78	1	1	0	A/A
control-778	1	65	1	1	0	G/A
control-779	1	74	0	0	1	A/A
control-780	1	75	1	0	1	A/A
control-781	1	55	0	0	0	G/A
control-782	2	68	0	0	1	A/A
control-783	2	59	0	0	1	G/G

control-784	1	59	0	0	0	A/A
control-785	1	59	1	0	0	A/A
control-786	1	56	0	0	1	A/A
control-787	2	63	0	0	1	A/A
control-788	1	70	1	1	0	G/A
control-789	2	71	0	0	1	A/A
control-790	1	52	1	0	1	A/A
control-791	2	68	0	0	1	A/A
control-792	2	53	0	0	1	A/A
control-793	1	61	1	1	0	A/A
control-794	1	74	0	0	1	A/A
control-795	2	72	0	0	1	A/A
control-796	1	74	0	0	0	A/A
control-797	2	68	0	0	0	G/A
control-798	1	76	0	0	1	A/A
control-799	1	61	0	0	0	G/A
control-800	1	61	0	1	1	A/A
control-801	1	74	1	0	1	A/A
control-802	1	71	1	0	1	A/A
control-803	1	56	0	0	0	A/A
control-804	1	64	1	1	0	A/A
control-805	2	53	0	0	1	A/A
control-806	1	71	0	0	1	A/A
control-807	1	52	0	0	1	A/A
control-808	1	70	0	0	0	A/A
control-809	1	52	1	0	1	A/A
control-810	1	66	0	0	0	A/A
control-811	2	57	0	0	0	A/A
control-812	1	56	1	1	0	A/A
control-813	1	75	1	1	0	?
control-814	2	62	0	0	0	G/A
control-815	1	74	0	0	1	A/A
control-816	2	70	0	0	0	G/A
control-817	1	73	0	0	0	A/A
control-818	2	61	0	0	0	A/A
control-819	1	73	0	0	1	G/A
control-820	2	57	0	0	0	A/A
control-821	2	63	0	0	1	A/A
control-822	2	58	0	0	1	G/A
control-823	1	66	0	0	0	A/A
control-824	1	67	1	0	1	A/A
control-825	1	65	1	1	1	G/A
control-826	1	50	0	0	0	A/A
control-827	2	55	0	0	1	A/A
control-828	1	74	0	0	1	A/A
control-829	1	56	0	0	1	A/A
control-830	2	59	0	0	0	A/A
control-831	1	65	1	1	0	G/G
control-832	2	72	0	0	0	G/A
control-833	2	47	0	0	1	G/A
control-834	1	58	1	1	0	A/A
control-835	1	60	1	1	0	A/A
control-836	1	69	0	0	1	A/A
control-837	2	62	0	0	0	G/A
control-838	1	74	1	1	1	A/A
control-839	2	61	0	0	1	A/A
control-840	2	69	0	0	1	A/A
control-841	1	78	0	0	0	G/A

control-842	1	70	1	0	1	A/A
control-843	2	53	0	0	0	A/A
control-844	1	58	0	0	1	A/A
control-845	2	61	0	0	0	A/A
control-846	1	68	0	0	0	G/A
control-847	1	68	0	0	0	A/A
control-848	1	69	0	0	1	A/A
control-849	1	61	0	0	0	A/A
control-850	2	53	0	0	0	A/A
control-851	1	69	0	0	1	A/A
control-852	2	45	0	0	1	A/A
control-853	1	55	1	1	0	A/A
control-854	2	52	0	0	1	G/A
control-855	1	67	0	0	1	A/A
control-856	1	53	1	0	1	G/A
control-857	2	59	0	0	1	A/A
control-858	1	49	1	1	0	A/A
control-859	2	59	0	0	1	G/A
control-860	1	55	1	0	1	G/G
control-861	1	62	0	0	1	A/A
control-862	2	63	0	0	1	A/A
control-863	1	57	0	1	0	A/A
control-864	1	53	0	0	0	A/A
control-865	2	68	0	0	0	A/A
control-866	1	76	0	0	0	A/A
control-867	2	79	0	0	0	A/A
control-868	1	45	0	0	1	A/A
control-869	1	72	0	0	0	G/A
control-870	1	67	1	1	0	A/A
control-871	1	52	0	0	1	G/A
control-872	2	47	0	0	0	A/A
control-873	1	53	0	0	1	G/A
control-874	2	56	0	0	0	G/A
control-875	1	70	1	0	1	G/A
control-876	1	70	0	0	1	A/A
control-877	2	53	0	0	0	A/A
control-878	1	52	0	1	0	A/A
control-879	1	63	0	0	0	A/A
control-880	2	67	0	0	1	G/A
control-881	2	48	0	0	1	A/A
control-882	1	61	0	1	0	A/A
control-883	2	71	0	0	0	A/A
control-884	1	44	0	0	1	A/A
control-885	1	69	1	0	0	A/A
control-886	1	59	0	0	0	A/A
control-887	1	70	1	0	0	A/A
control-888	1	68	0	0	0	G/G
control-889	1	52	0	0	1	A/A
control-890	2	65	0	0	1	A/A
control-891	2	48	0	0	0	A/A
control-892	2	59	0	0	0	G/A
control-893	1	69	1	1	0	A/A
control-894	1	50	0	0	0	A/A
control-895	1	70	0	0	0	A/A
control-896	2	53	0	0	1	A/A
control-897	1	70	1	1	0	A/A
control-898	1	71	1	0	0	A/A
control-899	1	54	0	0	1	A/A

control-900	1	56	0	0	1	A/A
control-901	2	58	0	0	1	A/A
control-902	1	79	0	0	1	A/A
control-903	2	72	0	0	0	A/A
control-904	1	60	0	0	0	A/A
control-905	2	73	0	0	1	A/A
control-906	1	60	1	0	0	A/A
control-907	2	57	0	0	1	A/A
control-908	1	55	0	0	0	A/A
control-909	2	68	0	0	0	G/A
control-910	2	61	0	0	1	A/A
control-911	1	45	1	1	0	G/A
control-912	2	64	0	0	0	A/A
control-913	1	76	0	0	1	A/A
control-914	2	66	0	0	0	G/A
control-915	2	67	0	0	0	A/A
control-916	2	51	0	0	1	A/A
control-917	1	69	0	0	0	A/A
control-918	2	67	0	0	1	A/A
control-919	1	73	1	0	0	A/A
control-920	1	75	0	0	1	G/A
control-921	1	51	1	0	0	A/A
control-922	1	65	1	0	0	A/A
control-923	2	61	0	0	1	G/A
control-924	2	65	0	0	0	A/A
control-925	2	70	0	0	1	G/A
control-926	1	65	0	0	1	G/A
control-927	1	60	1	0	1	A/A
control-928	1	58	1	1	1	G/A
control-929	2	71	0	0	0	A/A
control-930	2	67	0	0	0	A/A
control-931	2	66	0	0	1	A/A
control-932	1	68	0	0	1	A/A
control-933	1	43	0	0	1	A/A
control-934	1	70	0	0	0	A/A
control-935	2	47	0	0	1	A/A
control-936	2	52	0	0	1	G/A
control-937	1	52	0	0	1	A/A
control-938	2	61	0	0	0	A/A
control-939	2	66	0	0	0	A/A
control-940	2	59	0	0	0	A/A
control-941	1	63	1	0	1	G/A
control-942	1	59	1	1	0	A/A
control-943	1	72	1	0	1	A/A
control-944	1	70	1	0	0	G/A
control-945	2	56	0	0	1	A/A
control-946	2	69	0	0	1	A/A
control-947	1	58	1	1	1	A/A
control-948	1	49	1	1	1	G/A
control-949	2	60	0	0	1	A/A
control-950	1	51	1	1	0	A/A
control-951	2	51	0	0	1	A/A
control-952	1	75	0	0	0	G/A
control-953	1	46	1	0	1	A/A
control-954	1	64	1	0	0	A/A
control-955	1	72	1	1	0	A/A
control-956	1	75	0	0	0	G/G
control-957	1	56	1	1	1	A/A

control-958	2	46	0	0	0	A/A
control-959	2	56	0	0	0	A/A
control-960	2	72	0	0	1	A/A
control-961	1	79	0	0	1	A/A
control-962	1	53	0	0	1	A/A
control-963	1	68	1	1	0	A/A
control-964	1	69	1	1	0	A/A
control-965	1	70	1	0	1	A/A
control-966	1	75	1	0	1	G/A
control-967	2	66	0	0	1	A/A
control-968	1	67	0	0	0	A/A
control-969	1	67	0	0	1	A/A
control-970	2	54	0	0	1	A/A
control-971	1	53	0	0	0	A/A
control-972	1	65	1	0	1	A/A
control-973	2	60	0	0	0	G/A
control-974	2	48	0	0	0	A/A
control-975	1	52	0	0	0	A/A
control-976	2	62	0	0	0	A/A
control-977	2	61	0	0	0	G/A
control-978	1	56	0	0	1	A/A
control-979	1	57	0	0	1	G/A
control-980	1	61	1	1	0	A/A
control-981	2	65	0	0	0	A/A
control-982	1	69	0	0	0	A/A
control-983	1	75	1	1	0	A/A
control-984	2	68	0	0	0	A/A
control-985	1	60	1	0	0	A/A
control-986	1	65	0	0	1	G/A
control-987	2	56	0	0	0	A/A
control-988	1	50	0	1	0	A/A
control-989	1	49	0	0	0	A/A
control-990	2	47	0	0	1	A/A
control-991	2	71	0	0	1	G/A
control-992	1	71	0	0	1	A/A
control-993	1	69	0	0	0	A/A
control-994	1	74	0	0	0	A/A
control-995	2	64	0	0	0	A/A
control-996	2	72	0	0	0	A/A
control-997	1	70	1	0	1	A/A
control-998	1	71	1	1	1	A/A
control-999	2	61	0	0	1	G/A
control-1000	2	60	0	0	0	A/A
control-1001	2	66	0	0	1	A/A
control-1002	1	72	1	0	1	A/A
control-1003	1	69	1	0	0	G/A
control-1004	2	75	0	0	1	A/A
control-1005	2	43	0	0	1	G/A
control-1006	1	75	1	1	0	A/A
control-1007	2	66	0	0	1	G/A
control-1008	1	58	0	0	0	A/A
control-1009	1	67	0	0	1	G/A
control-1010	1	58	0	1	1	A/A
control-1011	1	62	0	0	1	A/A
control-1012	1	67	0	0	0	A/A
control-1013	1	67	1	1	0	G/A
control-1014	1	73	1	0	0	G/A
control-1015	2	66	0	0	1	G/A

control-1016	1	72	0	0	1	A/A
control-1017	2	61	0	0	0	A/A
control-1018	1	66	1	0	0	A/A
control-1019	1	74	0	1	0	A/A
control-1020	2	61	0	0	0	G/A
control-1021	2	70	0	0	1	A/A
control-1022	2	76	0	0	1	A/A
control-1023	1	70	1	0	1	A/A
control-1024	1	73	0	0	0	A/A
control-1025	1	75	0	1	0	G/A
control-1026	1	71	0	0	1	A/A
control-1027	1	69	1	1	1	A/A
control-1028	2	72	0	0	1	A/A
control-1029	2	75	0	0	1	A/A
control-1030	1	60	0	0	0	A/A
control-1031	2	72	0	0	1	A/A
control-1032	1	68	0	0	1	A/A
control-1033	1	56	0	0	1	G/A
control-1034	1	66	0	0	0	A/A
control-1035	2	51	0	0	0	A/A
control-1036	1	70	1	1	1	A/A
control-1037	1	70	1	0	0	A/A
control-1038	1	47	1	1	1	A/A
control-1039	2	72	0	0	0	A/A
control-1040	2	74	0	0	0	G/A
control-1041	2	59	0	0	0	A/A
control-1042	1	69	0	0	1	A/A
control-1043	1	74	0	0	0	A/A
control-1044	1	64	0	0	0	A/A
control-1045	2	66	0	0	0	A/A
control-1046	1	71	1	1	1	A/A
control-1047	2	72	0	0	1	A/A
control-1048	1	65	0	1	1	A/A
control-1049	2	67	0	0	1	A/A
control-1050	2	59	0	0	0	G/A
control-1051	2	53	0	0	0	A/A
control-1052	1	66	1	1	1	G/A
control-1053	2	75	0	0	1	A/A
control-1054	1	50	0	0	1	A/A
control-1055	1	67	0	0	1	G/A
control-1056	2	50	0	0	0	G/A
control-1057	1	69	0	1	1	A/A
control-1058	2	69	0	0	0	A/A
control-1059	1	63	1	0	0	A/A
control-1060	1	52	0	0	0	G/A
control-1061	2	69	0	0	0	A/A
control-1062	1	75	0	0	0	G/A
control-1063	1	76	1	0	0	A/A
control-1064	1	71	1	0	1	A/A
control-1065	1	61	0	1	0	A/A
control-1066	1	73	0	0	1	A/A
control-1067	2	68	0	0	0	A/A
control-1068	1	61	1	1	1	G/A
control-1069	2	61	0	0	0	G/A
control-1070	1	73	1	0	1	A/A
control-1071	1	62	0	0	1	A/A
control-1072	1	52	1	0	0	A/A
control-1073	1	62	1	1	1	G/A

control-1074	2	53	0	0	1	A/A
control-1075	1	74	0	0	0	A/A
control-1076	1	58	1	1	0	A/A
control-1077	2	74	0	0	1	A/A
control-1078	2	67	0	0	1	A/A
control-1079	2	83	0	0	0	G/A
control-1080	1	76	0	0	1	A/A
control-1081	1	44	1	0	0	G/A
control-1082	2	73	0	0	0	A/A
control-1083	1	49	0	0	0	A/A
control-1084	1	70	1	0	1	A/A
control-1085	2	64	0	0	0	A/A
control-1086	1	73	1	1	1	A/A
control-1087	1	73	0	1	1	G/G
control-1088	2	61	0	0	1	A/A
control-1089	1	65	1	1	1	A/A
control-1090	1	76	0	0	0	A/A
control-1091	1	69	1	0	1	G/A
control-1092	1	76	0	0	0	A/A
control-1093	2	68	0	0	1	A/A
control-1094	1	70	1	1	0	G/A
control-1095	1	64	1	1	1	A/A
control-1096	2	62	0	0	1	G/A
control-1097	1	64	0	0	1	G/A
control-1098	1	50	0	1	0	G/A
control-1099	1	46	0	0	1	A/A
control-1100	1	51	1	1	1	A/A
control-1101	1	59	0	0	0	A/A
control-1102	1	71	0	0	0	A/A
control-1103	1	74	0	0	1	G/A
control-1104	1	70	0	0	1	G/A
control-1105	1	69	0	0	1	A/A
control-1106	1	68	1	0	1	A/A
control-1107	1	58	0	1	1	A/A
control-1108	1	72	1	0	1	A/A
control-1109	1	66	1	0	1	A/A
control-1110	1	70	0	0	0	A/A
control-1111	2	48	0	0	0	A/A
control-1112	1	76	1	1	0	G/A
control-1113	1	61	0	0	1	A/A
control-1114	1	67	0	0	1	A/A
control-1115	1	75	0	0	1	G/G
control-1116	1	61	0	1	1	G/A
control-1117	1	74	0	1	1	A/A
control-1118	1	74	0	0	0	A/A
control-1119	1	61	1	0	1	A/A
control-1120	1	62	0	1	0	A/A
control-1121	1	72	1	1	1	A/A
control-1122	1	60	1	0	1	G/A
control-1123	1	72	0	0	1	A/A
control-1124	1	73	1	0	0	G/A
control-1125	1	83	0	0	0	A/A
control-1126	1	73	0	1	1	A/A
control-1127	2	41	0	0	0	A/A
control-1128	1	71	0	0	1	G/A
control-1129	1	58	1	0	1	G/G
control-1130	1	61	1	1	0	G/A
control-1131	2	49	0	0	0	A/A

control-1132	1	72	1	0	0	A/A
control-1133	1	72	0	0	1	A/A
control-1134	2	66	0	0	0	A/A
control-1135	1	68	0	0	0	A/A
control-1136	1	62	1	0	0	A/A
control-1137	2	45	0	0	0	G/A
control-1138	1	71	0	1	0	G/A
control-1139	1	59	0	0	1	G/A
control-1140	2	45	0	0	1	A/A
control-1141	1	71	0	0	1	G/A
control-1142	1	61	0	0	0	G/G
control-1143	1	66	1	1	0	A/A
control-1144	2	46	0	0	1	A/A
control-1145	2	67	0	0	0	G/A
control-1146	1	52	0	0	1	A/A
control-1147	1	65	1	0	0	A/A
control-1148	1	53	0	0	0	A/A
control-1149	2	65	0	0	0	A/A
control-1150	1	64	1	1	1	G/A
control-1151	1	61	1	1	1	A/A
control-1152	1	71	1	0	0	G/A
control-1153	1	75	0	0	1	A/A
control-1154	2	74	0	0	0	A/A
control-1155	1	58	1	1	1	A/A
control-1156	2	62	0	0	0	A/A
control-1157	1	57	1	0	1	A/A
control-1158	1	75	0	0	0	A/A
control-1159	1	64	1	0	0	A/A
control-1160	2	55	0	0	0	A/A
control-1161	2	59	0	0	1	A/A
control-1162	2	59	1	1	1	A/A
control-1163	1	74	1	1	0	G/A
control-1164	2	57	0	0	1	G/A
control-1165	1	59	1	0	0	G/A
control-1166	1	59	0	0	1	A/A
control-1167	2	58	0	0	1	A/A
control-1168	2	72	0	0	1	A/A
control-1169	1	71	1	1	0	A/A
control-1170	1	76	0	0	1	A/A
control-1171	1	60	1	1	0	A/A
control-1172	2	64	0	0	1	A/A
control-1173	1	60	0	0	0	A/A
control-1174	1	75	1	0	0	A/A
control-1175	1	53	0	0	0	A/A
control-1176	1	63	0	0	1	G/A
control-1177	1	46	0	0	1	A/A
control-1178	1	44	0	0	1	A/A
control-1179	1	66	1	0	0	A/A
control-1180	1	74	1	0	0	G/G
control-1181	1	53	1	1	0	G/A
control-1182	2	74	0	0	0	A/A
control-1183	2	62	0	0	0	G/A
control-1184	2	63	0	0	0	A/A
control-1185	1	74	0	0	1	G/A
control-1186	1	51	1	1	1	A/A
control-1187	1	58	0	1	0	G/A
control-1188	1	57	0	0	1	G/A
control-1189	2	56	0	0	1	A/A

control-1190	1	67	0	0	1	A/A
control-1191	1	67	1	1	1	G/A
control-1192	1	57	0	0	0	A/A
control-1193	1	54	0	0	1	A/A
control-1194	1	52	0	0	0	A/A
control-1195	2	52	0	0	0	A/A
control-1196	1	53	0	0	0	A/A
control-1197	1	59	0	0	0	A/A
control-1198	1	61	0	0	0	G/A
control-1199	2	**66**	0	0	0	A/A
control-1200	2	71	0	0	0	A/A
control-1201	1	63	0	0	0	A/A
control-1202	1	57	0	0	1	G/A
control-1203	1	67	0	0	0	G/A
control-1204	1	65	0	0	1	G/A
control-1205	1	56	0	0	1	G/A
control-1206	1	67	0	0	0	A/A
control-1207	1	71	1	1	0	A/A
control-1208	1	65	0	0	0	A/A
control-1209	1	55	0	0	0	A/A
control-1210	1	68	0	0	1	A/A
control-1211	2	53	0	0	0	A/A
control-1212	2	65	0	0	0	A/A
control-1213	2	72	0	0	0	G/A
control-1214	1	65	1	0	1	A/A
control-1215	1	65	1	0	0	A/A
control-1216	2	68	0	0	1	A/A
control-1217	1	62	0	0	1	A/A
control-1218	1	52	0	0	1	G/A
control-1219	1	70	1	1	0	A/A
control-1220	1	59	0	0	1	A/A
control-1221	1	71	0	0	1	A/A
control-1222	1	64	0	0	0	G/A
control-1223	1	56	0	0	1	A/A
control-1224	1	58	0	0	0	A/A
control-1225	2	49	0	0	1	A/A
control-1226	1	52	0	0	1	A/A
control-1227	2	56	0	0	0	A/A
control-1228	1	72	0	0	0	A/A
control-1229	1	48	0	0	0	G/A
control-1230	1	64	1	1	0	G/A
control-1231	2	53	0	0	0	A/A
control-1232	1	49	0	0	0	A/A
control-1233	1	61	1	0	0	A/A
control-1234	2	54	0	0	0	G/A
control-1235	1	57	1	0	0	A/A
control-1236	1	58	1	0	1	A/A
control-1237	2	68	0	0	0	G/A
control-1238	2	49	0	0	1	A/A
control-1239	2	61	0	0	0	G/A
control-1240	2	59	0	0	0	A/A
control-1241	2	57	0	0	0	A/A
control-1242	2	71	0	0	1	G/A
control-1243	1	55	0	0	1	A/A
control-1244	1	61	1	1	0	A/A
control-1245	1	53	0	0	1	G/A
control-1246	1	59	1	0	1	A/A
control-1247	2	61	0	0	0	G/A

control-1248	2	70	0	0	1	A/A
control-1249	1	69	1	0	0	A/A
control-1250	2	52	0	0	1	A/A
control-1251	2	58	0	0	0	G/A
control-1252	1	69	0	0	0	A/A
control-1253	1	63	0	0	0	G/A
control-1254	2	64	0	0	0	A/A
control-1255	2	59	0	0	1	A/A
control-1256	1	63	0	0	1	A/A
control-1257	1	63	1	0	0	A/A
control-1258	2	58	0	0	0	A/A
control-1259	2	62	0	0	0	G/A
control-1260	1	69	1	0	0	A/A
control-1261	1	62	0	0	1	G/A
control-1262	2	63	0	0	1	G/A
control-1263	1	63	1	0	1	A/A
control-1264	1	72	0	0	0	A/A
control-1265	1	61	0	1	0	G/A
control-1266	2	59	0	0	0	G/A
control-1267	2	59	0	0	0	G/G
control-1268	1	64	0	1	1	A/A
control-1269	2	59	0	0	0	A/A
control-1270	1	69	0	0	1	A/A
control-1271	1	61	1	0	0	A/A
control-1272	1	60	0	1	0	A/A
control-1273	1	64	0	0	1	G/A
control-1274	1	67	0	0	1	G/A
control-1275	1	65	0	0	1	A/A
control-1276	1	59	1	0	1	A/A
control-1277	1	64	0	0	1	A/A
control-1278	1	63	1	1	1	A/A
control-1279	1	57	0	0	1	A/A
control-1280	1	67	1	0	1	A/A
control-1281	1	66	0	0	0	A/A
control-1282	1	69	0	0	1	A/A
control-1283	2	63	0	0	0	A/A
control-1284	2	60	0	0	1	A/A
control-1285	1	67	0	0	1	A/A
control-1286	1	69	0	0	1	A/A
control-1287	2	59	0	0	1	G/A
control-1288	1	70	0	0	0	A/A
control-1289	1	68	1	0	1	A/A
control-1290	1	65	0	0	0	A/A
control-1291	1	68	0	0	0	A/A
control-1292	1	71	0	1	1	A/A
control-1293	2	63	0	0	1	A/A
control-1294	1	72	0	0	0	A/A
control-1295	2	71	0	0	1	A/A
control-1296	2	59	0	0	1	A/A
control-1297	1	62	0	0	0	A/A
control-1298	2	62	0	0	0	G/A
control-1299	1	65	1	0	0	G/A
control-1300	1	70	1	1	0	A/A
control-1301	1	71	0	0	0	A/A
control-1302	1	72	0	0	1	G/A
control-1303	1	63	1	1	0	A/A
control-1304	2	56	0	0	0	G/A
control-1305	1	71	1	0	0	G/A

control-1306	1	60	0	0	0	G/G
control-1307	1	54	0	0	0	A/A
control-1308	1	58	0	0	1	G/G
control-1309	1	51	0	0	1	A/A
control-1310	1	55	0	0	0	A/A
control-1311	1	71	1	0	0	G/G
control-1312	1	59	0	0	1	A/A
control-1313	1	54	0	0	1	?
control-1314	2	55	0	0	1	A/A
control-1315	1	59	0	0	0	A/A
control-1316	1	54	0	0	0	A/A
control-1317	1	69	1	1	0	A/A
control-1318	2	62	0	0	0	G/G
control-1319	2	62	0	0	1	A/A
control-1320	1	54	0	0	0	A/A
control-1321	1	57	0	0	0	A/A
control-1322	1	52	0	0	0	?
control-1323	1	54	0	0	1	G/G
control-1324	1	56	0	0	1	A/A
control-1325	1	52	0	0	0	A/A
control-1326	1	50	0	0	0	A/A
control-1327	1	62	0	0	0	A/A
control-1328	1	40	0	0	1	A/A
control-1329	1	49	0	0	1	A/A
control-1330	1	53	0	0	1	G/G
control-1331	1	45	0	0	0	G/G
control-1332	1	48	0	0	0	A/A
control-1333	1	43	0	0	0	G/G
control-1334	1	61	0	0	1	A/A
control-1335	1	51	0	0	1	G/G
control-1336	1	69	0	0	1	A/A
control-1337	2	72	0	0	1	G/A
control-1338	1	55	0	0	1	A/A
control-1339	2	50	0	0	1	G/A
control-1340	2	56	0	0	1	A/A
control-1341	1	53	1	1	0	A/A
control-1342	2	55	0	0	0	A/A
control-1343	1	62	0	0	1	A/A
control-1344	2	64	0	0	1	A/A
control-1345	1	49	0	0	1	A/A
control-1346	1	63	0	0	0	A/A
control-1347	2	61	0	0	1	A/A
control-1348	1	51	0	0	1	A/A
control-1349	1	47	0	0	0	A/A
control-1350	1	49	0	0	0	G/A
control-1351	2	62	0	0	1	A/A
control-1352	1	40	0	0	0	A/A
control-1353	1	59	0	0	0	G/A
control-1354	1	49	0	0	0	G/A
control-1355	1	63	0	0	0	G/A
control-1356	1	49	0	0	1	G/A
control-1357	1	66	0	0	0	G/A
control-1358	1	42	1	1	0	A/A
control-1359	1	41	0	0	0	A/A
control-1360	2	56	0	0	1	G/A
control-1361	1	60	1	1	0	G/A
control-1362	1	65	1	0	0	A/A
control-1363	1	55	0	0	0	A/A

control-1364	1	63	1	1	0	A/A
control-1365	2	40	0	0	0	A/A
control-1366	1	50	0	1	0	A/A
control-1367	1	50	1	0	0	A/A
control-1368	1	52	0	0	1	A/A
control-1369	1	40	0	0	1	G/G
control-1370	1	52	1	0	0	G/A
control-1371	1	55	1	0	1	A/A
control-1372	1	56	1	0	1	A/A
control-1373	1	49	0	0	1	A/A
control-1374	2	53	0	0	1	G/A
control-1375	1	51	1	0	1	G/A
control-1376	1	53	1	0	1	A/A
control-1377	1	52	1	0	0	A/A
control-1378	1	54	0	0	1	A/A
control-1379	1	53	1	1	0	A/A
control-1380	2	55	0	0	0	A/A
control-1381	1	56	1	0	0	A/A
control-1382	1	53	1	0	1	G/A
control-1383	1	51	1	0	0	G/A
control-1384	1	47	1	0	1	A/A
control-1385	1	56	1	0	1	G/A
control-1386	1	43	1	0	1	A/A
control-1387	1	50	0	0	1	A/A
control-1388	1	47	0	0	0	G/A
control-1389	1	50	1	1	0	A/A
control-1390	1	49	0	0	1	G/A
control-1391	2	56	0	0	0	A/A
control-1392	1	56	1	0	0	G/A
control-1393	1	47	1	0	1	A/A
control-1394	1	52	0	0	0	G/A
control-1395	1	55	0	0	1	A/A
control-1396	1	53	0	0	1	A/A
control-1397	1	56	1	0	1	A/A
control-1398	1	52	1	0	1	G/A
control-1399	1	54	0	0	1	A/A
control-1400	1	47	1	0	1	A/A
control-1401	1	53	0	0	1	G/A
control-1402	1	42	1	0	1	A/A
control-1403	1	44	1	0	0	G/A
control-1404	1	52	1	1	1	G/A
control-1405	1	55	0	1	1	A/A
control-1406	2	51	0	0	1	A/A
control-1407	1	49	0	0	1	A/A
control-1408	1	46	0	0	1	A/A
control-1409	2	52	0	0	0	G/A
control-1410	2	56	0	0	0	A/A
control-1411	1	54	1	0	0	A/A
control-1412	1	51	1	0	0	A/A
control-1413	1	47	0	0	1	A/A
control-1414	1	52	1	0	1	A/A
control-1415	1	53	0	0	1	G/A
control-1416	1	50	1	0	0	G/A
control-1417	1	56	1	1	0	A/A
control-1418	1	51	1	0	0	A/A
control-1419	1	45	1	0	1	A/A
control-1420	1	47	0	0	0	A/A
control-1421	1	53	1	0	0	G/A

control-1422	1	50	1	0	1	A/A
control-1423	1	56	1	0	0	A/A
control-1424	1	49	0	0	1	A/A
control-1425	2	53	0	0	1	A/A
control-1426	1	55	0	0	0	A/A
control-1427	1	49	1	0	0	A/A
control-1428	2	56	0	0	0	A/A
control-1429	1	55	1	0	0	A/A
control-1430	1	49	1	0	1	G/A
control-1431	1	56	1	0	0	G/A
control-1432	1	51	1	0	1	A/A
control-1433	1	53	1	0	0	A/A
control-1434	1	41	0	0	1	A/A
control-1435	1	37	1	0	0	G/A
control-1436	1	53	1	0	1	A/A
control-1437	1	51	1	0	1	G/A
control-1438	1	56	1	0	0	?
control-1439	2	32	0	0	0	G/A
control-1440	1	40	1	0	1	G/A
control-1441	1	54	1	0	1	A/A
control-1442	1	55	0	0	1	A/A
control-1443	1	45	1	0	0	A/A
control-1444	1	41	1	0	1	G/A
control-1445	1	54	1	0	1	G/A
control-1446	1	46	1	0	1	A/A
control-1447	2	50	0	0	1	A/A
control-1448	1	42	1	0	1	A/A
control-1449	1	55	1	0	0	A/A
control-1450	1	56	1	0	0	A/A
control-1451	1	53	1	1	0	G/A
control-1452	1	48	1	0	1	G/A
control-1453	2	56	0	0	0	G/A
control-1454	1	53	0	0	1	A/A
control-1455	1	55	1	0	1	A/A
control-1456	1	54	1	0	1	A/A
control-1457	1	53	0	0	1	G/A
control-1458	1	54	1	0	1	G/G
control-1459	1	51	1	0	0	A/A
control-1460	1	53	1	0	1	G/A
control-1461	1	52	1	0	0	A/A
control-1462	1	53	1	0	1	A/A
control-1463	1	50	1	0	1	G/A
control-1464	1	53	0	0	0	A/A
control-1465	1	53	1	0	1	A/A
control-1466	1	54	0	0	1	A/A
control-1467	1	47	1	0	0	A/A
control-1468	1	55	0	0	1	A/A
control-1469	1	55	1	0	0	A/A
control-1470	1	44	1	0	1	A/A
control-1471	1	54	1	0	1	G/A
control-1472	2	50	0	0	1	A/A
control-1473	2	52	0	0	1	G/A
control-1474	1	53	1	0	1	A/A
control-1475	1	52	1	0	0	A/A
control-1476	1	50	0	0	0	A/A
